# Circuits Regulating Pleasure and Happiness—Mechanisms of Depression

**DOI:** 10.3389/fnhum.2016.00571

**Published:** 2016-11-10

**Authors:** Anton J. M. Loonen, Svetlana A. Ivanova

**Affiliations:** ^1^Department of Pharmacy, University of GroningenGroningen, Netherlands; ^2^GGZ WNB, Mental Health HospitalBergen op Zoom, Netherlands; ^3^Mental Health Research Institute, Tomsk National Research Medical Center of the Russian Academy of SciencesTomsk, Russia; ^4^National Research Tomsk Polytechnic UniversityTomsk, Russia

**Keywords:** depression, stress, mechanism, basal ganglia, amygdala, habenula, neuroplasticity

## Abstract

According to our model of the regulation of appetitive-searching vs. distress-avoiding behaviors, the motivation to display these essential conducts is regulated by two parallel cortico-striato-thalamo-cortical, re-entry circuits, including the core and the shell parts of the nucleus accumbens, respectively. An entire series of basal ganglia, running from the caudate nucleus on one side, to the centromedial amygdala on the other side, controls the intensity of these reward-seeking and misery-fleeing behaviors by stimulating the activity of the (pre)frontal and limbic cortices. Hyperactive motivation to display behavior that potentially results in reward induces feelings of hankering (relief leads to pleasure). Hyperactive motivation to exhibit behavior related to avoidance of misery results in dysphoria (relief leads to happiness). These two systems collaborate in a reciprocal fashion. In clinical depression, a mismatch exists between the activities of these two circuits: the balance is shifted to the misery-avoiding side. Five theories have been developed to explain the mechanism of depressive mood disorders, including the monoamine, biorhythm, neuro-endocrine, neuro-immune, and kindling/neuroplasticity theories. This paper describes these theories in relationship to the model (described above) of the regulation of reward-seeking vs. misery-avoiding behaviors. Chronic stress that leads to structural changes may induce the mismatch between the two systems. This mismatch leads to lack of pleasure, low energy, and indecisiveness, on one hand, and dysphoria, continuous worrying, and negative expectations on the other hand. The neuroplastic effects of monoamines, cortisol, and cytokines may mediate the induction of these structural alterations. Long-term exposure to stressful situations (particularly experienced during childhood) may lead to increased susceptibility for developing this condition. This hypothesis opens up the possibility of treating depression with psychotherapy. Genetic and other biological factors (toxic, infectious, or traumatic) may increase sensitivity to the induction of relevant neuroplastic changes. Reversal or compensation of these neuroplastic adjustments may explain the effects of biological therapies in treating depression.

## Introduction

The burden of depression in modern society is enormous. According to the definition of the often applied criteria of the American Psychiatric Association's Diagnostic and Statistical Manual of Mental Disorders (DSM), major depressive disorder (MDD) is highly prevalent, and patients with MDD are seriously disabled. In the Dutch NEMESIS-2 study, the lifetime prevalence of MDD, diagnosed according to DSM-IV criteria (APA, 2000), was 13.1% for men and 24.3% for women (De Graaf et al., 2010). This prevalence was measured in 18–64 year-old, ambulatory Dutch citizens; however, a significant proportion of individuals experience their first depressive episode after the age of 50 years (i.e., women after menopause and seniors). Therefore, the risk of experiencing depression over a given lifetime is probably much higher than the figures imply. This is interesting, because the lifetime prevalence is far lower for other mental disorders, including schizophrenia (0.3–1%), bipolar disorder (1.7% in high income countries), and borderline personality disorder (1.6% median population prevalence) (APA, 2013). Even specific anxiety disorders, like panic disorder (3.8%), occur far less frequently than MDD (De Graaf et al., 2010). This very high lifetime prevalence of MDD indicates something about its position in the spectrum of mental disorders, and probably also, about its pathogenesis.

Depression is a mood disorder, and mood is considered an emotion. It is sometimes stated that emotions are a highly developed phenomenon, displayed only by the most sophisticated organisms on the planet earth; i.e., *Homo sapiens*. This observation is typically used as a basis for proposing that only humans can experience a depressed mood, because the emotional state of “feeling depressed” is a unique human quality. Primitive animals are believed to be incapable of feeling sad or insecure. However, the authors do not agree with this notion. In fact, upon reviewing the evolution of the vertebrate forebrain, the conclusion emerged that emotions, like pleasure or happiness, were among the most primitive modes of perceiving the environment (Loonen and Ivanova, 2015). Specific cognitive functions arise from relatively new regions of the cerebral cortex, and emotions probably arise from phylogenetically old parts of the brain.

Recently, a new model was presented that distinguished between two types of cortico-ganglio-thalamo-cortical tracks, which could regulate intuitive (emotional) and skilled (cognitive) behaviors. These tracks were called the limbic and extrapyramidal circuits, respectively (Loonen and Ivanova, 2016a). The motivation for exhibiting these two types of behaviors was regulated by two re-entry circuits; including the nucleus accumbens shell (NAcbS) and the nucleus accumbens core (NAcbC), respectively. The NAcbS-containing circuit motivated a display of misery-fleeing behavior; the NAcbC-containing circuit motivated reward-seeking conduct.

The present study aimed to use this model to interpret some of the abundant experimental data on the pathogenesis of depressive disorders. Misery-fleeing and reward-seeking behaviors constitute essential conducts for every free-moving animal to maintain life and to produce offspring. Therefore, the system regulating these behaviors can be traced back to brain structures of the earliest vertebrate ancestors of humans (Loonen and Ivanova, 2015). Continuous adequate functioning of these systems depends upon the ability to adapt sufficiently to the often changing and sometimes harsh conditions of living. These requirements of the ever changing biotope present a great challenge to the system, because it must constantly adapt its functional capabilities. The biological mechanisms required to accomplish these adaptations comprise neurochemical, neuro-endocrine, neuro-immune, and neuro-physiological components. These mechanisms will be described here, according to five current theories that postulate different neurobiological backgrounds that give rise to mood disorders (Loonen, 2013a). According to one school of thought, the cumulative burden of this active process of adaptation (i.e., allostasis) might result in overload (McEwen, 2012, 2015), and when the system starts to malfunction, it causes symptoms of depression. However, it may also be hypothesized that the system overload results when the adaptation process overshoots the current needs. This situation will be explained by considering how humans have changed their habitat over the last two centuries.

## Philosophical starting points

Modern humans are currently living under conditions which are considerably different from our biotope, even compared to a few centuries ago. We should recognize that our bodily systems are not intended, and perhaps not very suitable, for keeping us safe and sound in modern times. In fact, humans are not supposed to live to ages that are normally observed in current times. In the year 1600 AD, only about 600 million individuals existed in the entire world (Loonen, 2012). Until the Middle Ages, life expectancy after birth was about 25 years (Bocquet-Appel and Bacro, 1997; Eshed et al., 2004; Nagaoka et al., 2006), and this increased to 45 years after 1900 AD (Mackenbach and Looman, 2013). The low life expectancy was probably due to the large risk of infant mortality, combined with death in young adulthood from accidents, epidemics, plagues, wars, and childbirth. Most humans probably died from infectious diseases (Miller and Raison, 2015). When a person was wounded in an accident or aggressive attack, the wound was often infected with bacteria, and serious infections ultimately led to death. In women, giving birth often caused death, frequently due to infections. Therefore, it is quite clear that, to survive as an individual and as a species, humans required a very good immune system, capable of conquering the many challenges of life under dangerous circumstances. The chances of staying alive were probably highest in individuals that could activate this immune system under all conditions that might foster a wound infection. Therefore, the immune response can be considered a primary component of a complex stress response.

At the same time, humans lived in very unhygienic conditions. However, although dirt harbors a multitude of germs, sustained exposure to dirt may have represented a survival advantage (Miller and Raison, 2015). The immune response that defends against pathogenic germs—which may eventually have become commensals—must have been suppressed during normal life, to prevent immune overactivation to the extent that it caused damage to the human body. Moreover, the presence of commensals probably prevented other pathological microbes from invading and causing serious infections (Miller and Raison, 2015). In addition, the abundance of dirt, bacteria, and fungi, prevented the immune system from attacking common proteins and other macromolecules that can cause allergies and autoimmune diseases. However, the low prevalence of autoimmune diseases was also due to genetic selection, because a child or adult could not survive a serious immunological disease in ancient times. Therefore, it could be concluded that, currently, our immune system may be over-reacting and perhaps even causing more diseases than it fights. One of these diseases might be depressive disorder.

In present times, clinical depression is so common that it may not be considered an illness. It could be concluded from foregoing reflections that, perhaps we should think of depression as the price humans must pay for improving the standard of living and for dramatically increasing life expectancy. Humans possess a multimodal mechanism, which can adapt the organism's misery-fleeing and reward-seeking behaviors to a major extent, when required to deal with specific challenges. However, survival challenges are no longer frequently encountered in modern human life. Consequently, our adaptation mechanisms may be out of place (overshooting the actual needs) and lead to counterproductive behavior; for example, exaggerated stress reactions or food/substance abuse. Continuous anxiety and addiction states are regularly accompanied by specific mood problems (Koob and Le Moal, 1997; Mineka et al., 1998; Koob and Volkow, 2010; Loonen et al., 2016). Moreover, we should start with the notion that, during evolution, when humans were living in a natural habitat, depression may have been a beneficial mechanism for survival. Inactivity and hiding are probably good strategies for survival, when the organism is vulnerable to being killed. Therefore, it may be more fitting to consider clinical depression a reaction, like fever, vomiting, or diarrhea, which can improve one's chances of staying alive under difficult circumstances. Finally, the high prevalence of depression may be considered to result from selection pressure. Although humans may no longer die from tuberculosis or pneumonia, clearly, other conditions will cause death in due time. Once we can effectively combat cardiovascular diseases and cancer, humans will begin to die from, perhaps, neurodegenerative disorders, like Parkinson's or Alzheimer's disease. Inevitably, all living organisms must die. Hence, disorders like depression will become more prevalent and also significantly more life-limiting. This will be evident by comparing current and future rates of depression using standard diagnostic criteria, the absence of which unfortunately limits our ability to track changes in the prevalence of depression prior to the second half of the twentieth century.

A diametrically opposite view is that depression may be a valuable mechanism for adapting to current circumstances. The human way of life is rapidly changing, but we should not expect the human body to change at the same rate. The process of adaptation may take time. Throughout history, the human species has frequently been capable of surviving a changing environment. Modern humans originated from a small group in South Kenya about 195,000 years ago (McDougall et al., 2005). Humans populated the Americas about 18,000 years ago, and disseminated from the Arctic Ocean to the Argentinean *Tierra del Fuego*. Moreover, the human ancestors that invaded Eurasia about 40,000 years ago must have encountered several of the most recent glacial periods. Therefore, major adaptations to changing living circumstances are not unusual for mankind. However, it should be realized that, in the current era, during only about two centuries, humans have radically changed their biotope from a rural to an urban environment, and human life expectancy has doubled. This rate of change was far greater than any prior rates of change. These rapid changes may induce unusually strong adaptation reactions within the human species, which lead to aberrant activity in the biological processes that control misery-fleeing and reward-seeking behaviors. As a consequence, rapid change might result in genetic selection, which, through a common biological mechanism, ensures the fittest individuals can predominate.

Hence, several potential mechanisms may explain why depression is currently highly prevalent. Depression may be considered an aberration of a mechanism that originally favored survival (retreat from unfamiliar or threatening circumstances), or it may be a necessary mechanism that allows the human species to adapt to dramatic changes in the biotope (self-assessment/criticism). Regardless of the etiology, depression can be thought of as part of a mechanism involved in the process of adapting one's behavior to life in a rapidly changing, sometimes hostile world. Therefore, we might expect the pathophysiological mechanisms underlying depression to be related to this adaptation process. Below, we will describe how this common mechanism can be reduced from 5 current theories on the mechanism of depression.

## Description of our model

Behavior can be considered a mechanism where the brain manages input to create a specific output, which enables the organism to adapt to changed circumstances within the biosphere. In humans, input from the senses is primarily translated within the cerebral cortex into a specific behavioral output. Sensory information is processed within the posterior cerebral cortex in a stepwise fashion (Loonen, 2013b). Specific information is integrated with other sensory information and transmitted from the primary sensory cortex to the secondary sensory cortex, from there to the association cortex, and so on. Within the anterior cerebral cortex, a similar diverging flow of information occurs, which leads to the activation of specific brain regions, e.g., the motor cortex. Apart from this stepwise processing, other fibers connect to more distant regions that run in parallel. Every neural connection is capable of learning, due to the characteristics of glutamatergic transmission, which can increase or decrease the sensitivity of connecting synapses by inducing long term potentiation (LTP) or long term depression (LTD). Therefore, the cortex can “learn” to transmit specific sensory information to a specific output unit via a “preferred” cortical tract. Accordingly, the cerebral cortex learns to interpret sensory information and produce a specific behavioral response.

**Figure 1:**
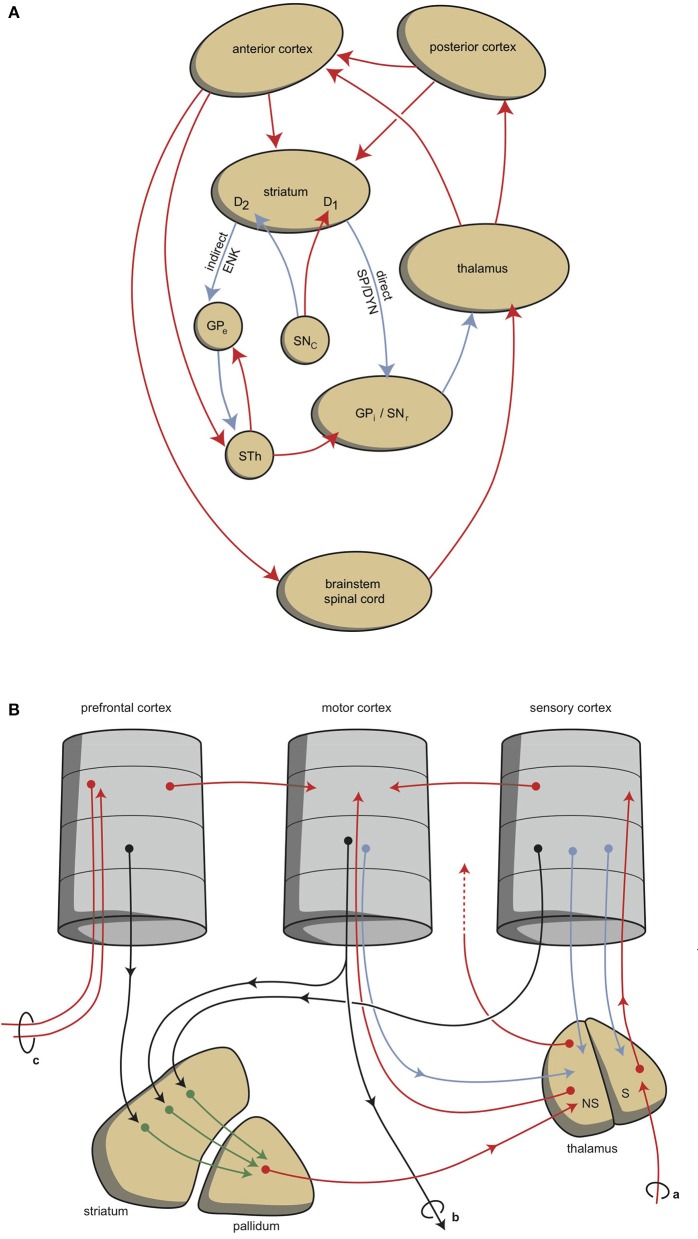
**Cortical-subcortical-processing units**. Extrapyramidal units are shown as an example of such cortico-striato-thalamo-cortical processing units. **(A)** Direct and indirect pathways lead to activation or inhibition of the anterior cortical endpoint. Abbreviations: D1, medium spiny neurons carrying dopamine D1 receptors; D2, medium spiny neurons carrying dopamine D2 receptors; DYN, dynorphin; ENK, enkephalin; GPe, globus pallidus externa; GPi, globus pallidus interna; SP, substance P; STh, subthalamic nucleus. Red arrows, excitatory; blue arrows, inhibitory. **(B)** Converging pathways through the basal ganglia correct the serially connected intracortical connections. Abbreviations: a, sensory input; b, motor output; c, to contralateral cortex; NS, non-specific part of the thalamus; S, specific part of the thalamus. Red, black, green, blue arrows, undetermined neurochemically.

Although this process is expedient, it can be expected to be highly sensitive to dysregulation, both in routine functions and in learning. Therefore, a parallel circuit has evolved, which includes subcortical structures. All processing units in the cerebral cortex also send information to the basal ganglia (Heimer, 2003). The route through the basal ganglia and thalamus leads to corresponding processing units in the anterior cortex (Loonen and Ivanova, 2013). This parallel circuit has stimulatory and inhibitory pathways and its glutamatergic synapses can also induce LTP and LTD (Figure 1A). Therefore, this parallel route through the basal ganglia enables the brain to correct serially transmitted information, when it arrives at the “final” destination. Moreover, the connection through the basal ganglia is convergent (Figure 1B; Alexander et al., 1986; Loonen and Ivanova, 2013; Loonen, 2013b). Hence, the processing units in the posterior and anterior cortices and their outputs converge within this subcortical circuit to the same output unit. Again, the “learning” ability of glutamatergic synapses within this framework make it possible to process a constantly varying input and produce very complex, sophisticated output patterns, in a reproducible, precise fashion.

This organization of connections is well-known as the extrapyramidal system, which regulates cognition and movements (Groenewegen, 2003). In our depression model, we suggest that a similar organization can be distinguished within the limbic cortex, although here, the structure is more complex and less modular, due to the ancient origins of these structures (Loonen and Ivanova, 2015, 2016a; Loonen et al., 2016). To simplify, we have concentrated on the primary limbic cortex within the superficial and deep corticoid regions of the amygdala. These regions are connected with many other archicortical and mesocortical areas. These corticoid regions can be considered input areas, and the centromedial (ganglionic or nuclear) region can be considered the output area of the amygdaloid complex. The stria terminalis connects the nuclear amygdala to the diencephalon, and from there, the anterior mesial frontal areas can be reached (Sewards and Sewards, 2003). However, the majority of output from the limbic basal ganglia flows to the brainstem. This connection is probably related to the fact that cortical motor centers were absent in the earliest vertebrate ancestors of humans (Loonen and Ivanova, 2015). In lampreys, movements are directed by diencephalic and brainstem motor centers (Loonen and Ivanova, 2015). In our opinion, in higher vertebrates, the nuclear amygdala represents the striatum of our oldest vertebrate ancestors. Nevertheless, the amygdala affects the motor output of higher vertebrates, including humans, by inducing the drive to seek food, warmth, comfort, etcetera, or to escape from pain, thirst, misery, etcetera (Sewards and Sewards, 2003).

Hence, two types of cortical-subcortical systems may be distinguished: extrapyramidal and limbic circuits. These systems have different first step relay stations; the extrapyramidal circuit includes the caudate nucleus, putamen, and nucleus accumbens core (NAcbC); the limbic circuit includes the nuclear amygdala, the connecting extended amygdala, the bed nucleus of the stria terminalis, and the nucleus accumbens shell (NAcbS). The extrapyramidal circuit regulates “rational,” cognitively constructed, skilled behavior, which is often goal-oriented and includes decision making. The limbic circuit regulates emotional (instinctive and automatic) behaviors, which are often defensive, and this regulation includes (attentive) salience. The two systems influence each other in a reciprocal (yin-and-yang-like) fashion; moreover, both systems can inhibit or activate, as the situation demands. It is generally accepted that the prefrontal cortex (PFC) is in control of selecting the appropriate response (Stuss and Knight, 2002; Fuster, 2008). The dorsolateral PFC is particularly important for controlling rational responses, and the ventromedial PFC controls emotional responses. Within the ventromedial PFC, the orbitofrontal cortex (OFC) plays a particularly noteworthy role, because it is essential for regulating the direction of motivation (Zald and Rauch, 2006).

Behavior can be a reaction to an influence in the environment, or it can also be generated by the individual. To enable a reactive or proactive behavior, motivation comes into play (Rolls, 1999; Stuss and Knight, 2002). Three stages of behavioral motivation can be distinguished: general motivation, initiative, and selective precedence-conveying (via inhibition). The OFC plays a significant role in regulating these processes by delivering input to the ventral striatum, the anterior cingulate cortex, and the amygdala.

**Figure 2:**
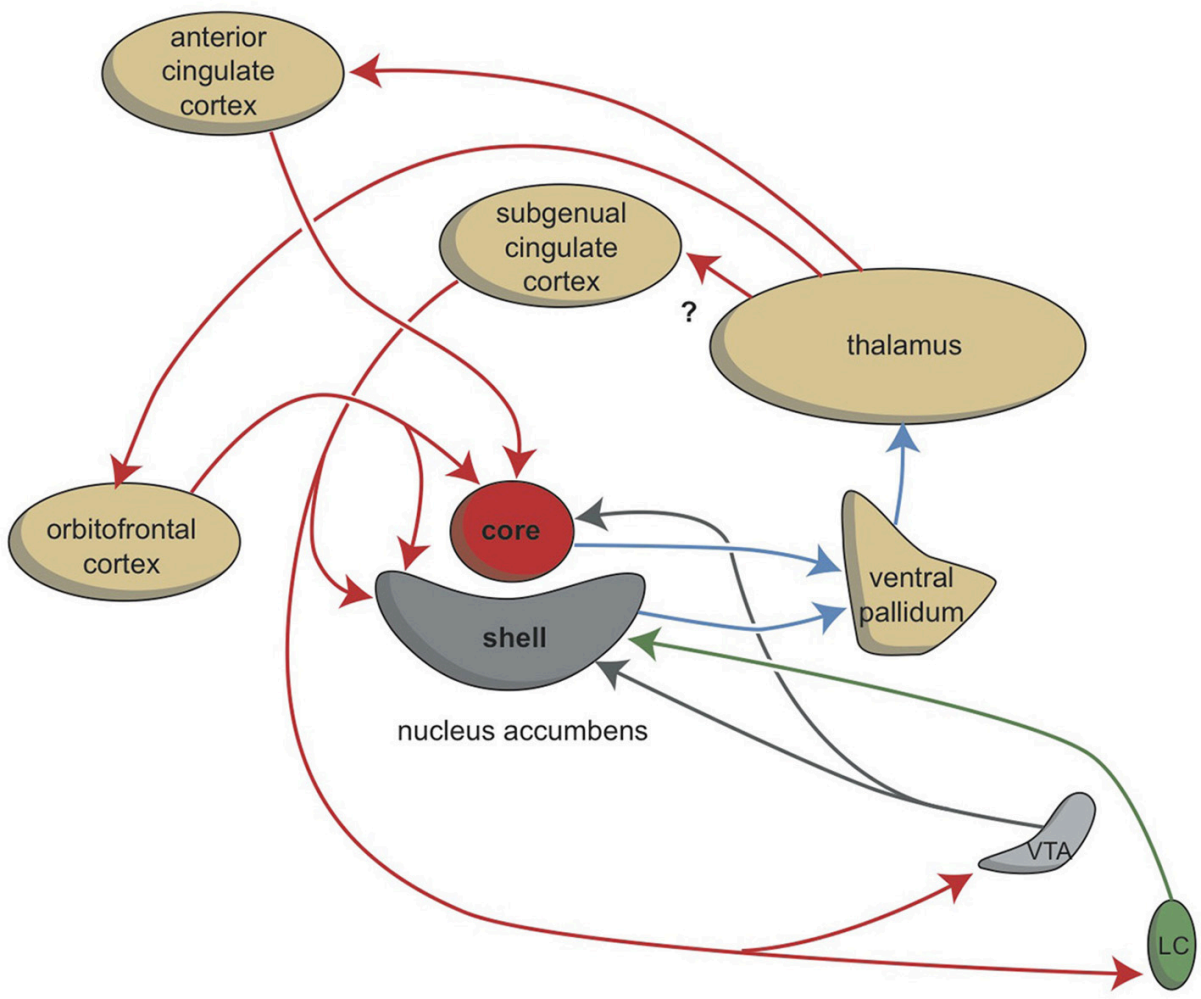
**Stimulation of the core and shell of the nucleus accumbens**. Adapted from Dalley et al. (2008). VTA, ventral tegmental area; LC, locus coeruleus. Red arrows, glutamatergic; blue arrows, GABAergic; gray arrows, dopaminergic; green arrow, adrenergic.

Although the extrapyramidal and limbic circuits regulate two different types of behavior (cognitive and intuitive, respectively), the individual must be highly motivated to express these conducts. This motivation requires the involvement of two specific structures: the NAcbC and the NAcbS (Figure 2; Groenewegen, 2007; Dalley et al., 2008; Loonen and Stahl, 2011). The NAcbC motivates the individual to show behavior that may lead to a feeling of reward. The NAcbS motivates the individual to show behavior that may lead to escape from misery (Loonen and Ivanova, 2016a). When high stimulation of these motivations suddenly ceases, the individual experiences feelings of pleasure (NAcbC) or feelings of happiness (NAcbS). Therefore, we distinguish between circuits that regulate pleasure and circuits that regulate happiness (Loonen and Ivanova, 2016a). In clinical depression, both circuits seem to be dysfunctional. Low activity in the reward seeking system results in the inability to experience pleasure (anhedonia), lack of energy, and indecisiveness. Particularly high activity in the misery-fleeing system results in continuous worrying, negative expectations, and dysphoric feelings.

The activities of the NAcbC and NAcbS, in turn, are regulated by monoaminergic nuclei within the midbrain. These nuclei transmit signals through dopaminergic (ventral tegmental area, VTA), adrenergic (norepinephrine, locus coeruleus), and serotonergic (raphe nuclei) tracts. In addition to their direct regulation of the NAcbC and/or NAcbS (Loonen and Ivanova, 2016a; Loonen et al., 2016), these monoaminergic nuclei regulate the activity of other, first relay-station, basal ganglia and important parts of other areas in the forebrain. Therefore, it may be concluded that behavioral output is controlled at three levels within the brain. The highest level is the cerebral cortex (isocortex, limbic cortex, corticoid amygdala, and hippocampal complex). The second level is the subcortical forebrain (dorsal striatum, ventral striatum, and extended amygdala). The third level of control is the midbrain (monoaminergic regulation centers).

**Figure 3:**
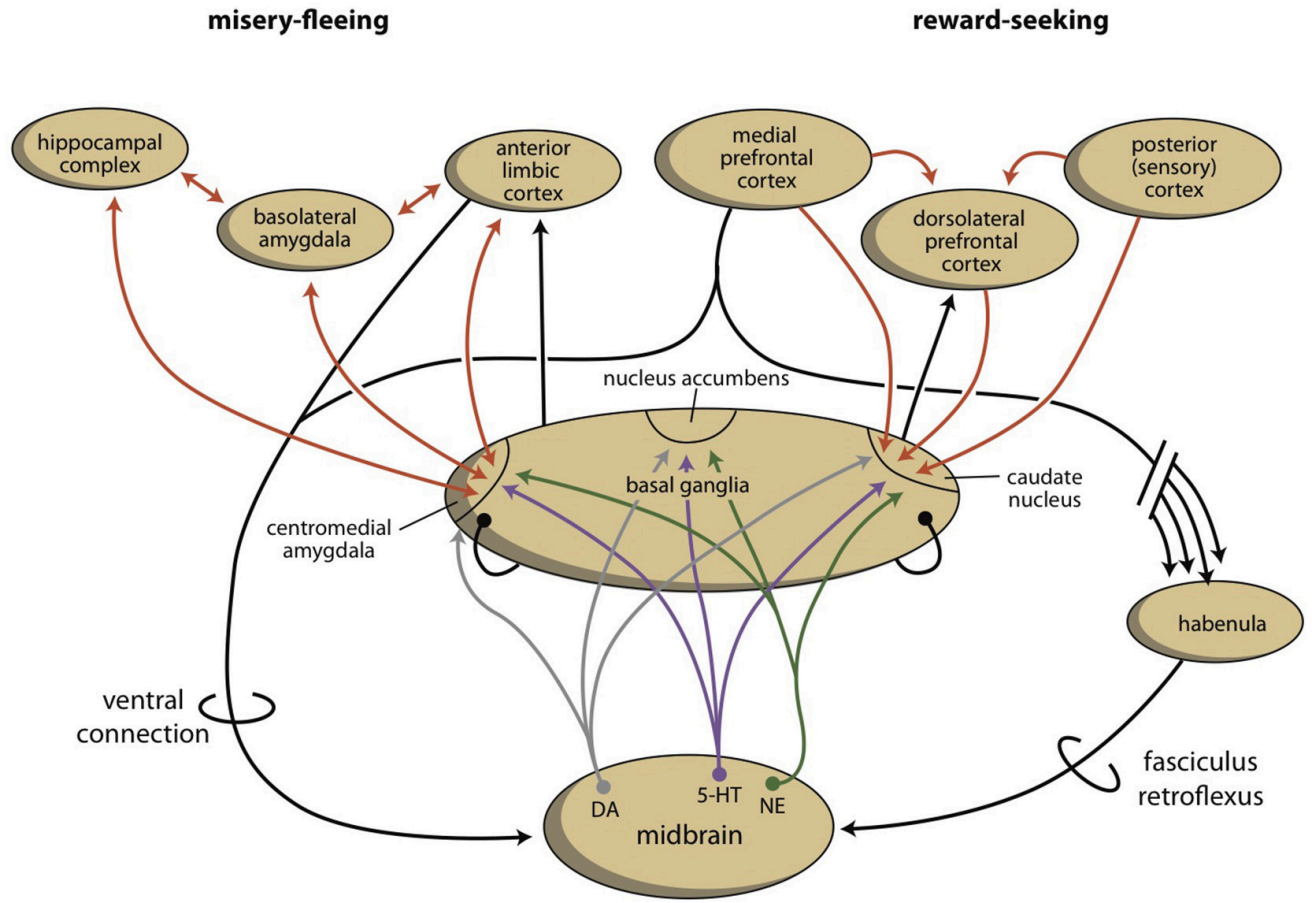
**The organization of the misery-fleeing and reward-seeking regulatory systems**. The series of limbic and extrapyramidal basal ganglia that form converging processing units are separated from each other by the system that contains the nucleus accumbens. These units regulate motivations to exhibit misery-fleeing and reward-seeking behaviors. The activity of the basal ganglia processing unit is regulated by monoaminergic neurons from the midbrain, which are, in turn, controlled by the cerebral cortex, through a direct (ventral) and an indirect (dorsal) connection. The dorsal connection includes the habenula and the fasciculus retroflexus. [Note: the figure is slightly changed in Loonen and Ivanova, (submitted)]. Red arrows, glutamatergic; gray arrows, dopaminergic; purple arrows, serotonergic; green arrows, adrenergic; black arrows, undetermined neurochemically.

As part of our depression model, we suggest that a fourth regulatory system exists, the habenula, which connects the cerebral cortex and midbrain systems (Figure 3; Loonen and Ivanova, 2016a; Loonen et al., 2016). Based on the regulation of appetitive behavior in lampreys, the lateral habenula has an important regulatory function (Loonen and Ivanova, 2015). In the lamprey, when a behavior is particularly rewarding, the lateral habenula promotes this behavior by intensifying stimulation of the phylogenetic homolog of the VTA. However, when the reward is smaller than expected or absent, the behavior is discouraged by inhibiting the VTA-equivalent in the lamprey. A similar mechanism of reward processing appears to be present in higher vertebrates (Matsumoto and Hikosaka, 2009; Bromberg-Martin et al., 2010; Zhao et al., 2015). The medial habenula has been shown to play a role in misery-fleeing behavior (Robertson et al., 2014; Viswanath et al., 2014). The habenula is part of the epithalamus, which also harbors the pineal gland and the stria medullaris. The habenula's projections to the midbrain were well conserved during vertebrate evolution (Hikosaka, 2010; Viswanath et al., 2014; Loonen et al., 2016). Its input structures are less certain in higher vertebrates, but they include important inputs from the septal areas and the amygdalar-hippocampal complex (Loonen and Ivanova, submitted). The latter inputs connect the habenula to the cerebral cortex. Moreover, a direct connection between the PFC and the monoaminergic midbrain centers affects the activity of the VTA (Loonen et al., 2016).

In conclusion, the extrapyramidal and limbic subcortical systems regulate cognitive (rational) and instinctive behaviors, respectively. The intensity of behavior that ultimately leads to reward is controlled by the cortico-striato-thalamo-cortical (CSTC) circuit that includes the NAcbC. The intensity of behavior that ultimately leads to safety is controlled by the CSTC circuit that includes the NAcbS. On the temporal side of the brain, the corticoid amygdala determines the appropriateness of flight, fight, or appetitive responses. Based on attentive salience, the nuclear amygdala initiates the proper emotional component of behavior. On the dorsal side of the brain, the caudate nucleus determines the suitability of the available repertoire of skilled behaviors; it selects the proper motor response to achieve the intended goals. The motivation to express these behaviors is regulated by monoaminergic centers within the midbrain. In turn, these monoaminergic centers are regulated by the cerebral cortex and corticoid amygdala through a dorsal connection that travels through the medial and lateral habenula. In particular, this latter mechanism is essential for adapting reward-seeking and misery-fleeing behavior to environmental changes. In the following overview, it will be shown that the neurobiological mechanisms involved in this process also appear to be included in current theories that explain the biological background of depression.

## Theories of depression

For many decades, the neurobiology of mood disorders has been the subject of intensive experimental and clinical research. Initially, the pharmacological effects of antidepressant agents were taken as a starting point for developing a theory of how depressive mood disorders arise. This resulted in the monoamine theory of depression. Most antidepressant drugs inhibit pre-synaptic transporters that are responsible for the reuptake of neurotransmitters from the synaptic cleft (Cooper et al., 2003; Leonard, 2003; Stahl, 2008). Tricyclic antidepressants (TCAs) inhibit the reuptake of norepinephrine and serotonin; in contrast, selective serotonin reuptake inhibitors (SSRIs) only inhibit serotonin reuptake (Maître et al., 1980). A few substances also affect the reuptake of dopamine (bupropion), but these are exceptions to the rule (Stahl, 1998; Feighner, 1999). A few antidepressants (mirtazapine) block pre-synaptic receptors and disinhibit the release of norepinephrine (Feighner, 1999). Psychostimulants also inhibit neurotransmitter reuptake, but in addition, they promote the release of neurotransmitters (Riddle et al., 2005). Psychostimulants also affect the release of dopamine. Classical monoamine oxidase inhibitors (e.g., tranylcypromine) decrease mitochondrial metabolism of the three primary neurotransmitters, serotonin, norepinephrine, and dopamine (Schildkraut, 1965; Coppen, 1967).

Other pharmacological effects of antidepressant treatments, in combination with the biochemical abnormalities observed in patients with depression, have led to the development of neuroendocrine and neuroimmunological theories of depressive mood disorders. An extrapolation of these ideas, in combination with the results of neuroimaging studies, led to the neuroplasticity theory. This latter theory is based on changes in neuroplasticity, but it also corresponds to findings from an electrophysiologically derived model, which is typically used to explain the increasing susceptibility of developing affective episodes in bipolar disorder. An entirely different approach emerged from the many circadian and seasonal aspects of depression. However, as we will discuss, this mechanism is not wholly different from the monoamine hypothesis, and it is firmly linked to the neuroendocrine and neuroimmunological theories. Therefore, five different theories can be distinguished: the monoamine hypothesis, the biorhythm hypothesis, the neuroendocrine hypothesis, the neuro-immune hypothesis, and the kindling (neurogenesis/neuroplasticity) hypothesis (Loonen, 2013a). We will briefly describe the backgrounds of these theories, with an eye to how they relate to the effects of neuroplastic changes, according to our depression model.

## The monoamine hypothesis

The oldest and most important theory on the pathogenesis of depression was developed in the 1960s and 1970s. That theory holds that depression is caused by a dysfunction in adrenergic or serotonergic neurotransmission (Van Praag, 1982; Baker and Dewhurst, 1985). Two observations formed the basis of this theory. First, it was observed that reserpine, an antihypertensive and antipsychotic drug, depleted monoamine neurotransmitters in the brain, and that this drug caused major depression in a significant proportion of patients that used it (Freis, 1954). The second observation was that, at that time, all known antidepressant drugs modified the levels of norepinephrine and serotonin (Schildkraut, 1965; Coppen, 1967). Later studies showed that cerebral spinal fluid levels of monoamines and their metabolites were quite different in patients with depression compared to individuals without depression (Schildkraut, 1965; Van Praag and Korf, 1971; Riddle et al., 2005). However, the main problem with this theory was that, although these drug-related changes occurred immediately after the initiation of treatment (Maître et al., 1980), the therapeutic effects occurred with a lag time of about 2 weeks. With the advent of the receptor binding technique, this lag time is currently thought to result from the fact that neurotransmitter receptor levels adapt slowly to pharmacologically-induced changes (Baker and Dewhurst, 1985). Chronic treatment with antidepressants were found to induce a decrease in the sensitivity of serotonin type-1A (5-HT1A) receptors in the hippocampus (Sibug et al., 1998; Subhash et al., 2000) and an increase in the sensitivity of β-adrenoceptors in the cerebral cortex (Sugrue, 1983).

The monoamine hypothesis arose from the observation that brain function depended on the existence of specific neurotransmitter systems (Nieuwenhuys, 1985; Nieuwenhuys et al., 2008). Here, we must distinguish between major and minor neurotransmitter systems (Loonen, 2013a,b). The vast majority of central nervous system (CNS) neurons secrete either glutamate (30%) or γ-aminobutyric acid (GABA; 50%) as neurotransmitters. These major transmitter systems are responsible for the evaluation and memorization of all sensory input. Moreover, these systems initiate, coordinate, and control all motor output from the brain. However, monoaminergic neurons, which secrete dopamine, norepinephrine, serotonin, or histamine as neurotransmitters, are far more important for neuropsychopharmacologists, even though these neurons comprise less than 2% of CNS neurons (Loonen, 2013a). This focus is probably related to the low acute toxicity of centrally-acting drugs, which act by interfering with monoamine receptors. For example, in the 1980s, patients with psychoses were treated with haloperidol, pimozide, or fluphenazine at daily dosages that corresponded to approximately 10,000 chlorpromazine equivalents (Andreasen et al., 2010), as part of a rapid neuroleptisation procedure (AJML, personal observation). Actually, some patients went swimming in spite of taking these extreme dosages, which must have blocked all available CNS dopamine receptors and may have also blocked serotonin, histamine, and norepinephrine transmission. These drugs were relatively safe, because they targeted minor systems, which are not directly responsible for evaluation and control, but smoothly regulate the activity levels of major neurotransmitter systems. Due to this low CNS toxicity, drug formulations that displayed unreliable pharmacokinetics or a non-specific pharmacodynamic profile could be used safely in treating patients with mental illnesses, which was certainly not possible with drugs that interfered to a large extent with glutamatergic neurotransmission.

The adrenergic and serotonergic systems have been intensively studied to explain the antidepressant effect of currently available pharmacotherapeutics. However, within the context of our depression model, the dopaminergic system is also critically important. As stated above, almost all antidepressants increase the concentration of norepinephrine and/or serotonin within the synaptic cleft. However, this is an acute effect, and it does not explain the lag time between the initiation of drug treatment and the start of clinical improvement. Shortly after the discovery of neurotransmitter binding sites, about four decades ago, it was found that chronic treatment with antidepressant drugs resulted in downregulation of β-adrenergic and 5-HT2 receptors (Sugrue, 1983). Since then, it has become clear that downregulation of postsynaptic β-adrenoceptors is a consistent and robust effect of chronic treatment with most antidepressants, and this effect is accelerated by co-treatment with SSRIs (Anand and Charney, 2000). Moreover, the serotonergic system, particularly the 5-HT2C receptor, seems to be involved in mediating antidepressant activity. The progressive downregulation of these receptors parallels the gradual onset of clinical effects observed with SSRIs (Millan, 2005). Hence, the acute effects of antidepressants are not believed to mediate their clinical activity; instead, clinical activity is due to the decreased sensitivities of β-adrenergic and 5-HT2C receptors, which are consequences of the acute effects.

### The adrenergic, dopaminergic, and serotonergic systems

**Figure 4:**
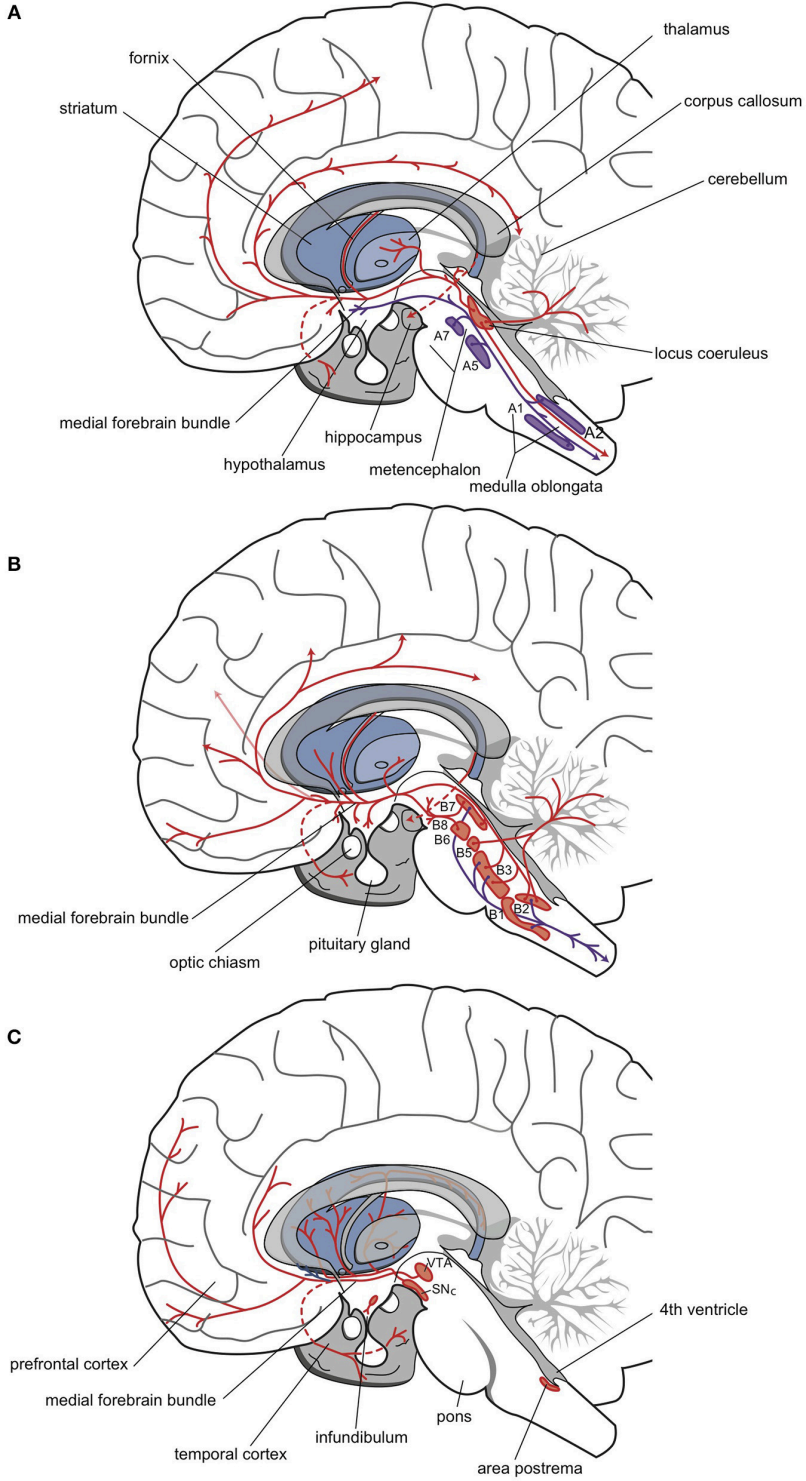
**Three monoaminergic neurotransmitter systems**. Adrenergic **(A)**, serotonergic **(B)**, and dopaminergic **(C)** neuropathways. Cell bodies are in the nuclei (red-filled shapes) positioned within the brainstem. Nerve fibers (red lines) terminate in the dorsal and ventral striata, amygdala, and frontotemporal cortex (Nieuwenhuys, 1985; Loonen, 2013b).

Norepinephrine was the first neurotransmitter thought to be involved in the mechanism of action of antidepressants. All cell bodies that produce this minor neurotransmitter are situated in the brain stem; about half are in the locus coeruleus complex, and the remainder are in a set of nuclei (A1, A2, A5, A7) of the autonomic nervous system (Figure 4A) (Nieuwenhuys, 1985; Nieuwenhuys et al., 2008; Loonen, 2013a,b). Fibers of these adrenergic neurons project to the diencephalon. The hypothalamus is primarily innervated by the group of “autonomic” brainstem nuclei (Nieuwenhuys, 1985). These projections are thought to be involved in, for example, the neuro-endocrine response (al-Damluji, 1993). Another set of adrenergic fibers runs from the brainstem, through the medial forebrain bundle, to more rostral parts of the forebrain (Figure 4A). The hippocampal complex, amygdala, and cerebral cortex are primarily innervated by adrenergic fibers that originate from the locus coeruleus (Nieuwenhuys, 1985). These fibers are believed to play important roles in arousal and impairments in PFC function during stress (Ramos and Arnsten, 2007).

In the 1970s, the involvement of the serotonergic system in the pathogenesis of depression was postulated by, among others, Van Praag (1982). The cell bodies of serotonergic neurons are located in the brain stem, and because they are found in the vicinity of the midline (the raphes), these nuclei are termed the upper and lower raphe nuclei (Figure 4B). The distribution of serotonergic fibers is somewhat more extensive than that of adrenergic fibers (Nieuwenhuys, 1985). Serotonergic fibers project to the spinal cord, several brain stem nuclei, the cerebellum, and the diencephalon (hypothalamus). Forebrain structures innervated by these fibers include the basal ganglia, the hippocampus, and the cerebral cortex (Nieuwenhuys, 1985). Seven types of serotonin receptors have been found, and all but one (5-HT3) are G-protein coupled receptors (Aghajanian and Sanders-Bush, 2002; Hoyer et al., 2002; Cooper et al., 2003; Leonard, 2003; Hannon and Hoyer, 2008; Loonen, 2013b). Most of these receptors are divided into several subtypes. Subtypes 5-HT1A, 5-HT2A, and 5-HT2C receptors have been most extensively studied for their role in depression. Stimulation of 5-HT1A receptors results in an increased influx of K+ ions and membrane hyperpolarization, which inhibits neurotransmission. Stimulation of 5-HT2A or 5-HT2C receptors has the opposite effect.

The dopaminergic system is thought to be primarily involved in psychosis and mania. However, in both mood disorders and schizophrenia, the dopaminergic system may also play a role in the development of depressive symptoms (particularly anhedonia) (Figure 4C; Loonen and Ivanova, 2016a). The midbrain (mesencephalon) contains three areas, A8, A9, and A10 (Nieuwenhuys, 1985). The latter two correspond to the substantia nigra pars compacta and the VTA. From there, dopaminergic fibers project to the dorsal and ventral striata, to the PFC, and to the mesial part of the temporal lobe. Previously, nigrostriatal (dorsal striatum), mesolimbic (ventral striatum), and mesocortical (cerebral cortex) projections were differentiated, but currently, these divisions are no longer distinguished (Nieuwenhuys, 1985).

### Monoamines and antidepressant activity

As described above, the adrenergic system influences the hypothalamus and the PFC. Depression is accompanied by a reduction in norepinephrine production and altered α2-adrenoceptor sensitivity, as reflected by the growth hormone response to clonidine (Anand and Charney, 2000). Because the hypothalamus is a controller of this partly endocrine response (Loonen and Ivanova, 2016a), it can be speculated that this feature of depression reflects a change in the ability of the hypothalamus to initiate a proper flight response. This deficiency may lead to the stimulation of compensatory mechanisms and result in hyperactivation of the misery-fleeing system. β-Adrenoceptors are predominantly located in the cerebral cortex, nucleus accumbens, and striatum (Pazos et al., 1985; Reznikoff et al., 1986; Arango et al., 1990; Joyce et al., 1992). By downregulating β-adrenoceptors in the NAcbS, antidepressants would dampen the misery-fleeing response by reducing its activity (Loonen and Ivanova, 2015, 2016a). However, modulating the effects of adrenoceptor stimulation within the PFC may also contribute to the antidepressant effects. To our knowledge, the precise role of β-adrenoceptors within the nucleus accumbens has not been extensively studied. However, serotonin is known to inhibit adrenergic function in the locus coeruleus complex (Stahl, 2009; Monti, 2011). Moreover, serotonin inhibits dopaminergic neurotransmission, both by inhibiting dopaminergic midbrain nuclei and by inhibiting dopamine release from terminal synapses, for example, in the PFC (Bonhomme and Esposito, 1998; Stahl, 2009; Morita and Nakayama, 2011; Loonen and Ivanova, 2016b). These inhibitory effects are mediated through excitatory 5-HT2A/2C receptors on fast-spiking GABAergic interneurons. However, there are important differences between 5-HT2A and 5HT2C receptors in their degrees of involvement in mediating serotonergic effects in the ventral and dorsal striata (Loonen and Ivanova, 2016b). It should be noted that SSRIs may augment parkinsonism in vulnerable patients (Leo, 1996; Gerber and Lynd, 1998). These effects are probably mediated by stimulating 5-HT2A/2C receptors, and they are counter-productive in alleviating depression. However, drugs like mirtazapine and trazodone, which are known to block these receptors, may exert antidepressant activity through the same mechanism (Millan et al., 2000; Stahl, 2009). Dopamine has an important stimulatory influence in the PFC (Arnsten, 2011). This may alleviate the lack of initiative (dorsolateral PFC) and activate circuits that motivate reward-seeking behaviors (mesial PFC and nucleus accumbens). However, dopamine is probably only important in a subset of patients, based on the limited efficacy of dopaminergic drugs (e.g., classical monoamine oxidase inhibitors) in “normal” depression and their high efficacy in depressions refractory to TCAs and SSRIs (Stahl and Felker, 2008).

Apart from their direct influences on the hypothalamus and PFC, serotonergic projections also affect temporal lobe structures, like the amygdaloid complex and hippocampus (Nieuwenhuys, 1985; Graeff et al., 1996; Berumen et al., 2012). In our model, an important overlap exists between the antidepressant and anti-anxiety effects of serotonergic drugs. Both effects are reported to be related to relief from a chronic stress-related state. Serotonergic fibers that originate in the dorsal and medial raphe nuclei regulate amygdalar activity by stimulating the 5-HT2 receptor family. However, the precise role of the 5-HT2A vs. the 5-HT2C receptor is not known (Bombardi, 2014). Moreover, the role of the 5-HT2B receptor has rarely been studied. The amygdala plays an important role in initiating the emotional response by activating the hypothalamus to execute, for example, anxiety/fear schemes (Aggleton, 2000; Inoue et al., 2011). The amygdala receives input from various brain structures (also unidentified sensory information), and it can react very quickly to this information in case of a possible threat. This rapid response is partly due to response conditioning (Pare and Duvarci, 2012; Maren et al., 2013). This input is transmitted from the sensory association cortex, the limbic cortex, and the parahippocampal region (Sinclair et al., 2009). The antidepressant effects of SSRIs may be related to the downregulation of 5-HT2C receptors. At the onset of SSRI treatment, indirect activation of 5-HT2C receptors participates in anxiogenic effects and the inhibition of sleep, sexual behavior, and appetite (Millan, 2005). This condition is known as the “jitteriness/anxiety syndrome” (Sinclair et al., 2009). Conversely, progressive downregulation of 5-HT2C receptors parallels the gradual onset of clinical SSRI efficacy (Millan, 2005). This downregulation may result in decreased sensitivity to fear-inducing or stress-inducing input and decreased activity in the limbic circuit, which may explain both the antidepressant and anxiolytic effects of SSRIs. We believe that these effects are mediated within the amygdaloid complex (Loonen and Ivanova, 2016a).

The hippocampal complex plays an important role as a controller of declarative memory formation (Eichenbaum, 2006; Cutsuridis and Wennekers, 2009). Within the hippocampal region, sensory information is identified as either something that occurred previously or something new and unexpected (Loonen, 2013b). Therefore, the hippocampal complex may have an important influence on the amygdala. The initiation of an emotional response is either facilitated with a warning (e.g., potential harmful situation) or inhibited with an assurance (e.g., innocent object, no threat). Thus, when a potentially harmful object is identified, the emotional response may be inhibited, then followed by facilitation of a cognitive defense reaction, when the hippocampal complex sends outputs to different parts of the PFC (Warburton and Brown, 2010; Euston et al., 2012). Serotonin has complex effects on hippocampal function. A chronic dysfunction of the hippocampus may result in a chronic state of hyperarousal and fear. Serotonin has two receptor effects that prevent hippocampal dysfunction: it directly stimulates 5-HT1A receptors (Ogren et al., 2008) and it modifies the effects of glucocorticoid receptors (GRs) (Erdeljan et al., 2001; Laplante et al., 2002). Stress and glucocorticoids are known to induce hypoplasia in the hippocampus, and the SSRI fluoxetine has been shown to protect hippocampal neurons against this influence (Czéh et al., 2007; Nagano et al., 2012). It is possible that both receptor effects are strongly interrelated. However, other biological responses may also result from the stimulation of 5-HT1A receptors (Ogren et al., 2008; Savitz et al., 2009). Very little is known about a possible influence of 5-HT2B receptors. Some studies suggest that 5-HT2B receptors may play a role in embryonic development (e.g., of the enteric nervous system) (Nebigil et al., 2001), and at least part of their effects may include modulating the activity of astrocytes (Hertz et al., 2015). In this context, the experiments of Diaz et al. (2012) are interesting. They found that genetic or pharmacological inactivation of 5-HT2B receptors could abolish some long-term behavioral and neurogenic SSRI effects and greatly attenuate the SSRI-induced increase in hippocampal extracellular serotonin concentrations. Conversely, direct agonists of 5-HT2B receptors mimicked these SSRI effects in the absence of SSRIs (Diaz et al., 2012). Hence, stimulation of 5-HT2B receptors may play a role in mediating the neurogenic effects of SSRIs. Serotonin is believed to promote adequate functions, or to suppress inadequate functions of the hippocampal complex. Either effect would result in inhibiting the amygdala, which would abolish an inadequate emotional response. As a result, the cognitive response would be allowed to take over. This would result in relieving an emotional disorder, whether a mood disorder or an anxiety disorder. This mechanism might explain the additive effects of cognitive behavioral therapy in combination with SSRIs in treating refractory depression (Wiles et al., 2013). Thus, SSRIs enhance the ability of the PFC to adopt cognitive schemes that limit stressful, depressive responses.

Based on the foregoing observations, it can be concluded that norepinephrine, serotonin, and dopamine may play important roles in the genesis of depressive disorders. The role of dopamine is restricted to a specific subset of depressive reactions, including TCA/SSRI refractory depression, depression in schizophrenia (caused by dysfunction of the PFC), depression in Parkinson's and Alzheimer's Diseases, and antipsychotic drug-induced depression. We propose that norepinephrine primarily exerts effects in the hypothalamus, PFC, and striatum (including the dorsal striatum and NAcbS), and that serotonin primarily exerts effects in the hippocampal complex/amygdala. In both cases, the effects increase the likelihood that an inappropriate response is suppressed and an appropriate response takes over. This explains the time needed for an actual change to occur. Thus, it is the basis for the lag in treatment effects.

## Biorhythm hypothesis

Several aspects of the symptomatology of depression indicate a malfunction in the biological clock (Wirz-Justice, 2006; McCarthy and Welsh, 2012). Depressive disorders are well known to be accompanied by sleep disturbances (Fleming, 1989; Thase, 1998; Tsuno et al., 2005; Srinivasan et al., 2009; Aizawa et al., 2013). In patients with a depressive disorder, the time period between the moment they fall asleep and the moment of detecting the first rapid eye movement (REM) episode, is remarkably shorter than in individuals without depression. In some individuals with depression, REM sleep occurs instantaneously. This phenomenon results in a remarkable distortion of the sleep architecture; i.e., the sequence of sleep stages during the night. Moreover, patients with depression experience interrupted sleep and late night insomnia. Apart from these sleep disturbances, other biorhythm disorders are evident in depression (Thase, 1998; Bunney and Bunney, 2000). In some patients, mood and other symptoms show typical fluctuations in severity over the day (Tölle, 1991). In the morning, they typically feel worse, and during the course of the day, things tend to improve slightly. Also other biorhythms become irregular, such as body temperature and the cortisol rhythm (Bunney and Bunney, 2000). Moreover, in some patients, depressive episodes show a typical seasonal pattern (Rosenthal et al., 1987; Melrose, 2015).

Different sleep disorders can be illustrated by comparing hypnograms between patients without or with depression, before and after treatment with electroconvulsive therapy (ECT) (Haffmans et al., 2006). The hypnogram of a patient in depression is entirely different from a normal hypnogram. The sleep phases are irregularly distributed, and the sequence of sleep phases is abnormal. After a patient recovers from depression, a normal hypnogram is observed (Haffmans et al., 2006; Loonen, 2013a).

**Figure 5:**
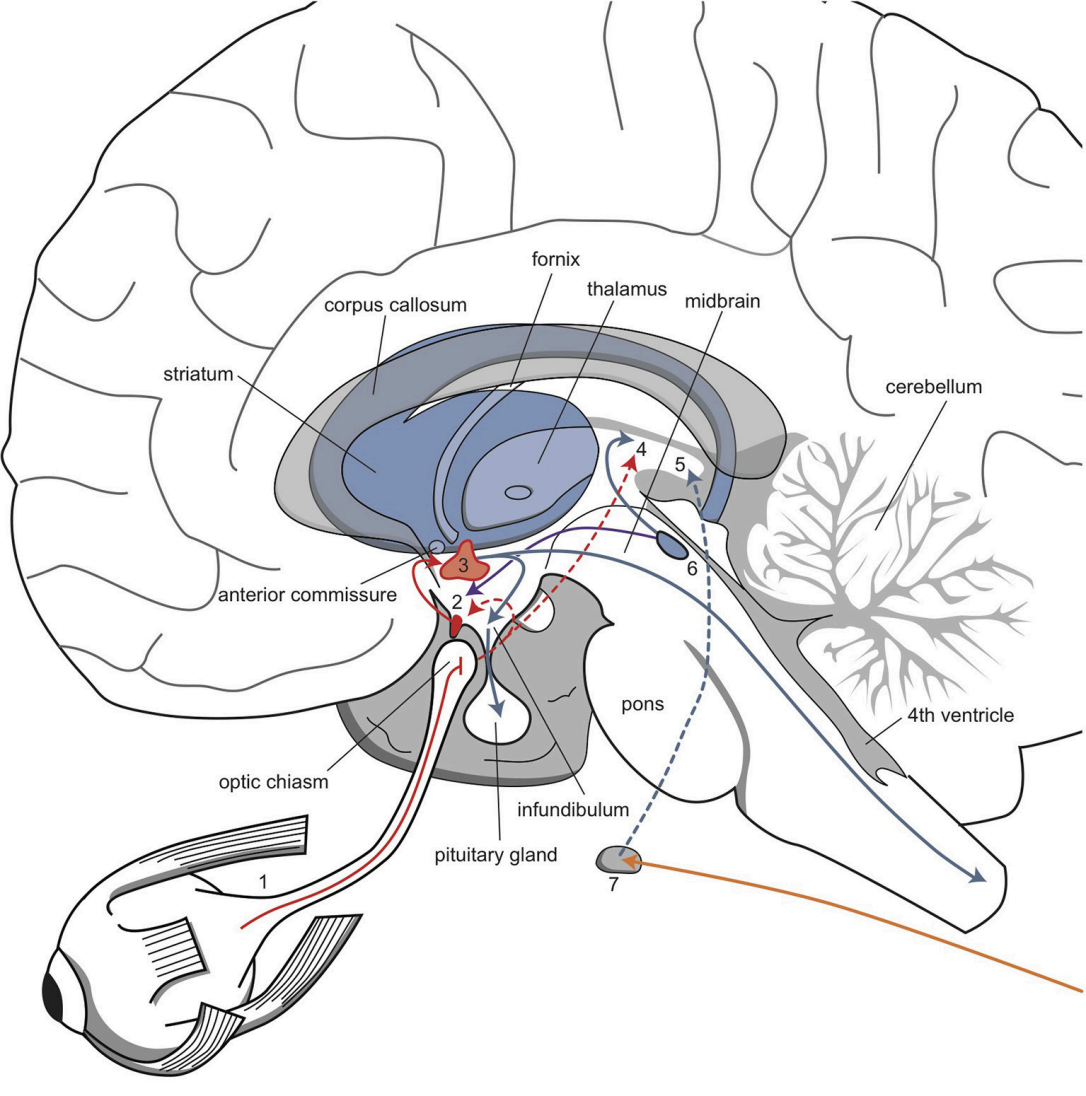
**The network that regulates circadian rhythms**. Retinal fibers are directly connected to the suprachiasmatic nucleus and thalamus. The suprachiasmatic nucleus is connected to the paraventricular nucleus, which influences endocrine and sympathetic activity. Sympathetic fibers from the superior cervical ganglion regulate melatonin secretion from the epiphysis. 1, retina; 2, suprachiasmatic nucleus; 3, paraventricular nucleus; 4, thalamus; 5, pineal gland; 6, raphe nuclei; 7, superior cervical ganglion. Red, orange, and blue arrows, undetermined neurochemically.

The described biorhythms depend on the function of several genes that regulate the endogenous biological clock (Bunney and Bunney, 2000; Wirz-Justice, 2009; McCarthy and Welsh, 2012). A circadian pacemaker in the brain comprises a group of approximately 10,000 neurons in the nucleus suprachiasmaticus (SCN) of the anterior hypothalamus. This pacemaker conducts, drives, and synchronizes all 24-h rhythms in the body (Wirz-Justice, 2009; McCarthy and Welsh, 2012). When isolated in culture, SCN neurons “tick” at an endogenous frequency, driven by constitutive clock genes (Wirz-Justice, 2009). Both the SCN and individual SCN cells have a circadian period, which is typically somewhat longer than 24 h in humans (Pagani et al., 2010). Thus, to synchronize to the 24-h day-night cycle, the SCN requires daily entrainment signals, so-called “Zeitgebers.” Daylight and melatonin play important roles in the entraining process. The interaction between daylight, melatonin, and the circadian rhythm is highly complex (Wirz-Justice, 2009; Bunney and Bunney, 2012); here, we will represent the entrainment process with a model that is highly simplified (and not entirely correct) (Loonen, 2013b). According to this model, the biological clock of the SCN is reset by full exposure to daylight, when the eyes are opened and daytime activity starts. The SCN is directly connected to the retina by fibers that deviate from the optic tract (Figure 5). Therefore, this mechanism also functions in cortical blindness, but not in retinal blindness. The sensitivity to entrainment by daylight is thought to be enhanced by melatonin in both the retina and the SCN. Melatonin is a hormone secreted by the pineal gland during sleep, provided that the sleep period matches the night phase of the circadian rhythm. Melatonin secretion is stimulated by fibers in a branch of the sympathetic nervous system that projects from the superior cervical ganglion to the pineal gland and by fibers that originate in the SCN. Melatonin binds to specific receptors in the SCN (Mel-1a) and in the retina (Mel-1b). These two locations explain why treatment with an evening dose of melatonin has therapeutic effects on biorhythms in patients with dysfunctional retinas (e.g., retinal blindness) (Skene and Arendt, 2007). Upon awakening, the presence of melatonin increases SCN entrainment by daylight. However, studies on melatonin and sleep have reported many contradictory findings.

The biorhythm hypothesis of depression postulates that depressive symptoms are caused by a dysfunction in the SCN that affects the generation of a correct circadian rhythm (Wirz-Justice, 2006; Bunney and Bunney, 2012; McCarthy and Welsh, 2012). One hypothesis says that, in depression, there is a mismatch between the biological rhythm and the true natural rhythm. When the biological rhythm is shorter than 24 h, the mismatch may cause a false start. It is possible that a false start might cause serious problems. However, contradictory results were reported from studies that aimed to establish a phase-advance in the overall 24-h pattern of body temperature in patients with depression (Bunney and Bunney, 2012). Another hypothesis holds that a disorder in entrainment causes depression, at least in some patients. This disorder may arise when daylight is insufficient to produce a “Zeitgeber.” In turn, this insufficiency may be related to insufficient retinal stimulation by daylight or insufficient sensitivity of the SCN, or both. A subpopulation of patients with depression experienced therapeutic effects with exposure to bright light, in addition to treatment with an evening dose of melatonin (Terman and Terman, 2005; Pail et al., 2011). However, many patients with depression do not respond to bright light treatment. Therefore, this mechanism is not likely to be the primary defect, and depression may not be comparable to a sort of exaggerated hibernation state in humans. However, the therapeutic results could suggest that, in a subpopulation of patients, this mechanism may play a role by inducing neuro-endocrine disturbances that are related to the pathogenesis of depression.

In addition to the SCN, the habenula may also play an important role in depression. The habenula has reciprocal connections with the pineal gland, and these structures have evolved in close association (Hikosaka, 2010; Loonen and Ivanova, 2015). The habenula plays a prominent role in sleep, particularly by regulating REM sleep (Hikosaka, 2010). Moreover, the habenula has important functions in reward-based decision making, avoidance of punishment, and behavioral responses to stress (Hikosaka, 2010). In some species, these processes may be closely linked to animal migration. The lateral habenula receives inputs from both the SCN and melanopsin retinal ganglion cells, which provide light entrainment signals to the circadian system (Benarroch, 2015). It is very possible that changes in the daily dark-light rhythm induce some animals to start migrating to improve the reward they perceive from daily living circumstances. In some animals, the habenula controls behavior that is induced by detecting changes in electric or magnetic fields, and these fields may play an important role in navigation (Hikosaka, 2010). The interaction between the SCN and the habenula may link the biorhythm hypothesis directly to our framework of the control of reward-seeking and misery-fleeing activities.

## Neuro-endocrine hypothesis

The neuro-endocrine hypothesis holds that depressive disorders result from dysregulation of the endocrine system. At least three endocrine subsystems have been postulated to play important roles in the pathogenesis of depressive disorders. One endocrine subsystem includes the thyroid gland and another subsystem includes the endocrine gonads. Each of these subsystems is known to function improperly in a variety of mood disturbances (Esposito et al., 1997; Tichomirowa et al., 2005; Studd and Nappi, 2012). Although relevant, discussing these subsystems can be considered a separate subject; therefore, we will not deal with them here in further detail.

In this review, we will concentrate on a third subsystem, the hypothalamic–pituitary–adrenal (HPA) axis (Fava, 1994; Merola et al., 1994; Stokes, 1995). Several HPA abnormalities are observed in mood disorders; however, they are not consistently observed, and they seem to be variable among different subgroups of patients with depression. It is evident that cortisol plays a multifaceted role. With depression, diurnal cortisol rhythms are disturbed, patients are resistant to the feedback action of exogenous glucocorticoids (e.g., dexamethasone), and basal cortisol levels may be elevated (Herbert, 2013). In addition, in several types of depression, patients do not respond to the administration of corticotrophin releasing hormone (CRH) with the expected increase in cortisol levels. These findings indicate that impaired corticosteroid receptor function may be the key mechanism in the pathogenesis of depression. This impairment results in dysfunctional stress hormone regulation. Specific tests were developed to detect compromised corticosteroid receptor function. The most sensitive test combined the administration of dexamethasone and CRH (Ising et al., 2005). These tests showed that the HPA axis exhibited hyperactivity and low sensitivity to feedback inhibition in patients with MDD. These changes are currently believed to be orchestrated by CRH.

Studies have demonstrated that, in depression, abnormal functions could be detected at the level of the hypothalamus (Raadsheer et al., 1994, 1995). CRH is secreted from hypothalamic paraventricular cells, and it stimulates the pituitary gland to produce ACTH; ACTH stimulates the cortex of the suprarenal glands to secrete cortisol into the general circulation. Finally, circulating cortisol inhibits CRH secretion from the hypothalamus and ACTH secretion from the pituitary gland, which represents an inhibitory feedback loop. This feedback loop appears to be disrupted in depression (Bao et al., 2008). In depression, the hypothalamic paraventricular cells display hyperplasia, and thus, they secrete elevated levels of CRH into the primary portal circulation of the pituitary gland. In response, the pituitary gland produces elevated ACTH, which then stimulates the adrenal glands. However, elevated cortisol levels do not appear to correct the levels of CRH and ACTH.

CRH is an important component of the complex emotional response initiated and controlled by the hypothalamus (Loonen and Ivanova, 2016a). In addition, CRH is an intracerebral neurotransmitter and/or neuromodulator, which was shown to cause an acute increase in the firing rate in the locus coeruleus (Anand and Charney, 2000). In turn, the high firing rate could result in stimulating the CSTC circuit that includes the NAcbS, which would increase the motivation to flee from misery-inducing stress.

Cortisol is secreted from the adrenal glands in hourly pulses. The largest pulse amplitude occurs at the beginning of the circadian activity period (Joëls et al., 2012; Gibbison et al., 2013). Cortisol can penetrate into the brain, where it has multiple effects, depending on the concentration (Joëls, 2006; Joëls et al., 2006, 2012). At low concentrations, cortisol induces recovery and repair, and it prepares the cerebrum for an adequate, rapid response to stressful situations, through nuclear (genomic) mineralocorticoid receptors (MRs). At high levels, cortisol modifies and optimizes the stress response. High cortisol normalizes brain activity some hours after exposure to a stressful event and promotes consolidation of the event for future use, through nuclear glucocorticoid receptors (GRs). However, high cortisol also directly activates the hippocampus and other limbic structures, which induces them to participate actively in the stress response, through membrane-associated (nongenomic) MRs (Joëls et al., 2006, 2012). Thus, high cortisol levels facilitate the emotional response and inhibit the cognitive response to stress. In addition, high cortisol levels protect CNS nerve cells against potential damage due to uncontrolled, heavy activity.

As mentioned, cortisol affects the hippocampus and other limbic areas (Joëls et al., 2012). These regions express two types of cortisol receptors; MRs and GRs. However, the mineralocorticoid, aldosterone, penetrates poorly into the brain, and its blood concentration is roughly 100-fold lower than cortisol concentrations (Joëls et al., 2012). Therefore, aldosterone probably rarely binds to cerebral MRs, and it is not likely to play a major role in the brain. In contrast, cortisol, a glucocorticoid, binds to both MRs and GRs, which are ligand-driven transcription factors that regulate genomic expression in the cell nucleus. Thus, MRs and GRs are called nuclear or genomic receptors (Joëls et al., 2012). MRs have a high affinity for cortisol, and they can bind to cortisol even at low concentrations; i.e., during the nadir/interpulse intervals of the cortisol secretion curve. Activation of MRs causes tonic inhibition in nerve cells, which maintains cell integrity and stability. Therefore, MR activation proactively controls the stress response; the cell is in a state of readiness. Apart from nuclear MRs, there are also membrane-bound MRs, with far lower affinity for cortisol (Joëls et al., 2006). Membrane MRs mediate rapid alterations in hippocampal excitability at the beginning of the stress response (Joëls et al., 2012).

GR receptors have low affinity for cortisol (Joëls et al., 2012). GR receptors are only activated when cortisol levels are high. This activation is not specific, because MRs are maximally activated under these conditions. Activation of the GR is particularly useful for ending the stress response. Thus, GR activation reactively controls the stress response. In addition, membrane-associated (nongenomic) GRs are also activated. Within the hypothalamus and amygdala, activation of membrane GRs suppresses neuronal activity (Joëls et al., 2012). Within the hypothalamic paraventricular nucleus, this effect is probably related to a rapid feedback mechanism.

In patients with a depressive disorder, the function of this mechanism is altered (Lucassen et al., 2006; Joëls, 2011). It has been postulated that sustained hyperactivity in the HPA-axis and a MR/GR imbalance, precipitated by (early) life stress, may generate a phenotype that is vulnerable to depression (De Kloet et al., 2005). Thus, when the functions of MRs and GRs are imbalanced in the hippocampus, hypoplasia may occur, and the consequence is hippocampal dysfunction (De Kloet et al., 2005; Lucassen et al., 2006). When the hippocampus fails to form declarative memories and cannot identify objects and events, the emotional fear response will dominate the picture.

A few experimental findings have indicated that the antidepressant effects of drugs, particularly SSRIs, are related to the effects of cortisol on the hippocampus. Stimulating 5-HT1A receptors protected the hippocampus against cortisol-induced dysfunction and cortisol-induced hypoplasia (McAllister-Williams et al., 1998; Wang et al., 2009). Also, chronic SSRI-mediated stimulation of 5-HT1A receptors appeared to repair the MR/GR imbalance (Lai et al., 2003; Zhou et al., 2014). This repair could restore the ability of sensory information to inhibit the amygdala appropriately, which removed the apparent danger perceived, and placed the individual at ease.

The foregoing descriptions should be considered an oversimplification, because the hippocampus is not a homogeneous brain area. A converging body of evidence has indicated that the hippocampus is functionally dissociated along its septo-temporal axis; the dorsal section is more involved in learning/memory and spatial navigation, and the ventral region is more linked to emotional behavior and regulation of the neuroendocrine stress axis (Tanti and Belzung, 2013). Animal models of depression exhibit effects restricted to the ventral hippocampus; in contrast, SSRIs act more uniformly on dorsal and ventral adult neurogenesis (Tanti and Belzung, 2013). Moreover, cortisol also directly affects the corticoid amygdala and the medial PFC (Joëls et al., 2012). These regions may also be targeted by serotonergic drugs.

It should be emphasized that hypovolemia of the hippocampus is not necessarily related to the same mechanism involved in the dysfunction associated with depressive disorder. In an excellent review article, Czéh and Lucassen (2007) argued that hippocampal shrinkage was probably not due to either a decline in neuronal development in the dentate gyrus or an increase in neuronal cell death. In fact, hippocampal hypovolemia may primarily result from fluid loss, due to a shift of fluid between the extracellular fluid compartment and cerebrospinal fluid or blood. This problem remains to be elucidated.

Despite the fact that neurogenesis and morphological changes (e.g., dendritic shrinkage) make small contributions to changes in hippocampal volume, these factors may be important for function. Most studies have been conducted in animal models, particularly rodents, where morphological changes are probably far more elaborate than in primates, including humans. However, despite these limitations, the neuroplastic changes observed most likely reflected important functional changes (Fuchs et al., 2006; Masi and Brovedani, 2011; Suri and Vaidya, 2013).

This brings us back to the role of serotonin. We previously hypothesized that the fear-flight response was initiated by the amygdala and controlled by the hypothalamus (Loonen and Ivanova, 2016a). The amygdala sends a signal to the hypothalamus to start or increase an emotional fear/flight reaction, in response to sensory information received in the corticoid part of this complex. This process is facilitated or inhibited by information stored in the hippocampus through the hippocampal identification and memorization process (episodic memory). After identification, the hippocampus sends an inhibitory signal to the amygdala and medial habenula and a stimulatory signal to the PFC, to ensure an adequate, cognitively planned response.

In addition to this mechanism, chronic stress induces direct neuroplastic changes in the medial PFC and in the amygdala (Fuchs et al., 2006). We will return to this phenomenon later.

In conclusion, many considerations support the notion that depression is related to inadequate biological stress management. This situation may result from an imbalance between GR and MR functions, particularly (but not exclusively) within the hippocampus. This imbalance may be reflected by neuroplastic alterations in hippocampal neurons. We believe that these alterations correlate with inappropriate interactions between the hippocampus and the amygdala, the hippocampus and the medial habenula, and possibly, also between the hippocampus and the PFC. This could result in inadequate selection of a depressive emotional response, which corresponds to hypermotivation to escape from exaggerated misery.

## Neuro-immune hypothesis

Before we discuss the neuro-immune hypothesis for the pathogenesis of depressive disorders, we must describe the influence of neurotrophic factors (Friedman, 2012). As mentioned above, cortisol has profound neuroplastic activity. Stimulation of corticoid receptors in the hippocampus resulted in neuroplastic alterations. This type of activity is exhibited by many neurotrophic factors, including some cytokines that are produced by immuno-competent cells, both inside and outside the brain (Licinio and Wong, 1997; Dantzer, 2007; Khairova et al., 2009; Capuron and Miller, 2011). In addition, the functions of the HPA axis and the immune system are intricately interconnected. Both systems can be considered essential constituents of the biological defense mechanism against sources of misery, like attacks from other living organisms (e.g., germs or predators). Also, both systems consist of mutually interacting biochemical and behavioral components. During the last two decades, it has also become evident that the CNS contains its own immune system, that this immune system is actively involved in stress reactions, and that an important interaction exists between the CNS and peripheral immune systems, on one hand, and between both immune systems and other CNS regulatory systems, on the other hand (Lampron et al., 2013). These interactions are separate from the role of microglia, which probably also regulate CNS functions in a non-inflammatory manner (Graeber, 2010; Kettenmann et al., 2013).

Neurotrophic or neural growth factors have profound actions on nerve cell functions (Friedman, 2012). They enhance or limit axon and dendrite sprouting and the formation of new neuronal branches and projections. They bind to specific receptors expressed on nerve and glia cells. Similar to peripheral neurons, new nerve cell branches in the CNS are believed to find their way to specific target cells by following chemotactic neurotrophic factors produced by specific neuroglia cells that function as “guidance” cells. Moreover, when a nerve cell makes contact with a neuron, specific neurotrophic factors modify the contact point to form a regular synapse. Also, synaptic activities are regulated by neurotrophic factors. In contrast, other neurotrophic factors inhibit specific synapses, induce regression of neuronal contacts and branches, and even induce apoptosis. Well known examples of neurotrophic factors include nerve growth factor (NGF) and brain-derived neurotrophic factor (BDNF), in addition to several neurotrophic cytokines (Friedman, 2012). Thus, cytokines, which are primarily associated with immune system function, comprise an important category of neurotrophic factors.

Cytokines are regulatory proteins that can act as autocrine, paracrine, or endocrine (humoral) factors (Dantzer, 2007) by binding to specific, high affinity, cell surface receptors. They are produced by different immune-competent cells and have pleiotropic effects (multiple actions and multiple target cells), including important effects in the CNS (Licinio and Wong, 1997; Miller et al., 1999). When immune activation occurs in peripheral organs, a signal is transmitted to the brain in several ways, including cytokines that cross the blood-brain barrier through saturable transporters, humoral transmission via the circumventricular organs, and neural transmission via the afferent nerves (e.g., the vagal nerve) that innervate the site in the periphery, where the inflammatory response occurred. This signal ultimately results in the synthesis and release of pro-inflammatory cytokines in the brain by activated astrocytes, microglia, vascular endothelial cells, and fibroblasts (Licinio and Wong, 1997; Miller et al., 1999; Dantzer, 2007; Capuron and Miller, 2011; Lampron et al., 2013). Cytokine receptors are present in the brain, and they are activated, in most cases, not by peripheral cytokines that enter the brain, but by cytokines that are produced locally in the brain in response to peripheral cytokines. These locally produced cytokines act on other glial cells, and ultimately, on neurons (Dantzer, 2007). In addition to their role in the elaboration of the central component of the acute phase inflammatory response, cytokines are also involved in brain development and neuronal plasticity (Dantzer, 2007). Some cytokines diminish the functional capacity of GRs, which directly contributes to insufficient glucocorticoid signaling. Cytokine administration *in vivo* and *in vitro* has been shown to reduce both GR number and function (Leonard and Myint, 2009).

**Figure 6:**
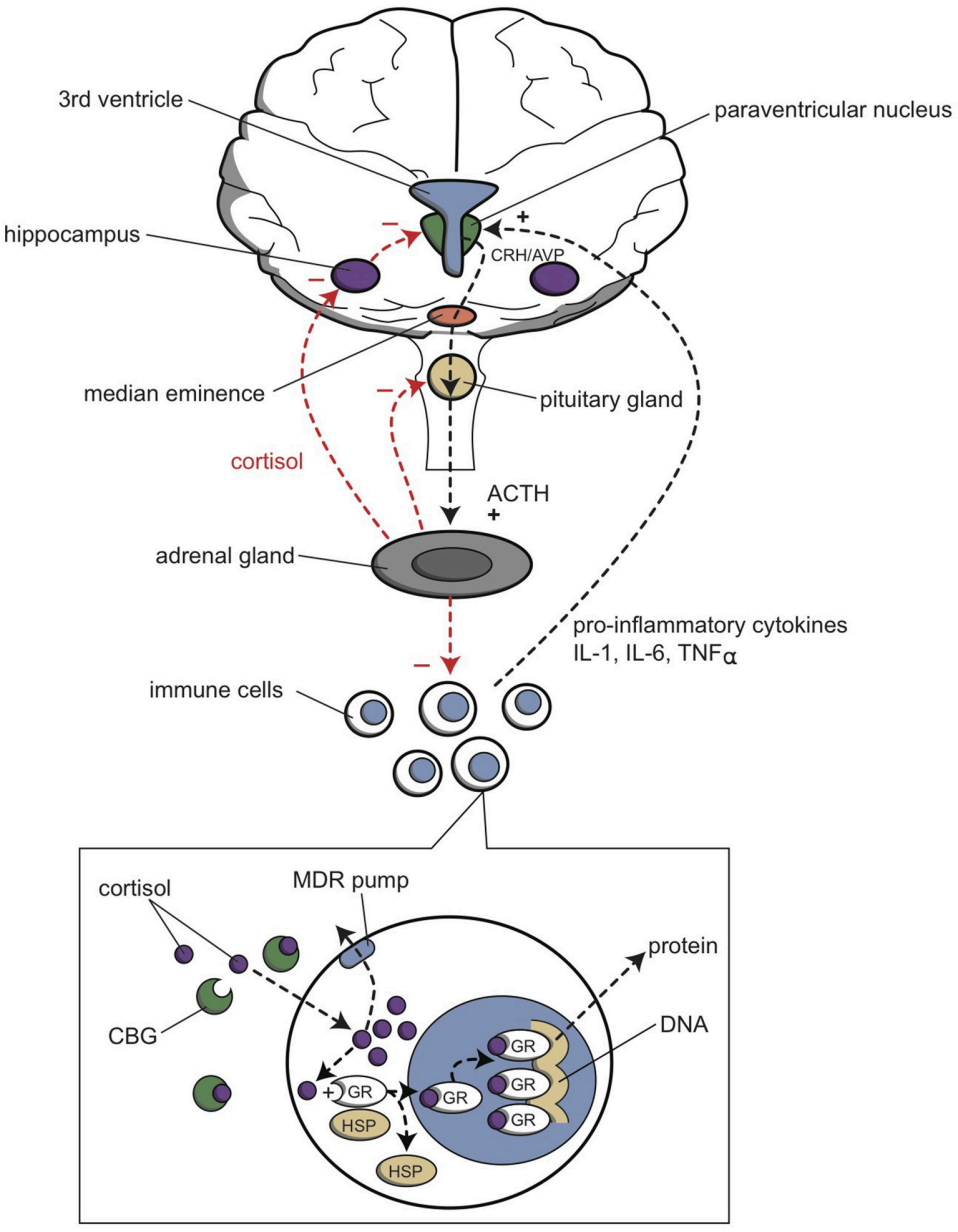
**Relationship between the HPA axis and the immune system**. The hypothalamic paraventricular nucleus (green) stimulates the production of cortisol from the adrenal gland (gray); cortisol inhibits the production of pro-inflammatory cytokines by immune cells (white and blue), by the mechanism shown in the inset. Cortisol also inhibits (−), and pro-inflammatory cytokines activate (+), the paraventricular nucleus. ACTH, adrenocorticotropic hormone; AVP, arginine vasopressin; CBG, cortisol binding globulin; CRH, corticotropin releasing hormone; GR, glucocorticoid receptor; HSP, heat shock protein; IL-1, interleukin 1; IL-6, interleukin 6; MDR pump, multidrug resistance protein (P-glycoprotein); TNFα, tumor necrosis factor alpha.

The functions of the HPA axis and the immune system are mutually dependent (Haddad et al., 2002; Haddad, 2008; Leonard and Myint, 2009; Miller et al., 2009). Cortisol inhibits CRH production in the hypothalamus and ACTH production in the pituitary gland (Figure 6). In the periphery, cortisol binds to intracellular receptors in peripheral immune cells. These cortisol/receptor complexes translocate to the nucleus and inhibit the immune response in two ways. First, they interact with other transcription factors to abolish cytokine production; second, they directly bind to DNA to stimulate gene transcription, which produces inhibitors that act as anti-inflammatory factors. On the other hand, certain pro-inflammatory cytokines, such as interleukin (IL)-1, IL-6, and tumor necrosis factor (TNF)-a, are potent stimulators of CRH, and therefore, they stimulate the HPA axis. These pro-inflammatory cytokines are produced in the periphery by a variety of immune cells, such as activated monocytes, macrophages, T-cells, B-cells, natural killer cells, and fibroblasts, in response to different stimuli, including pathogen-associated molecules, danger signals, and stress. It has been postulated that pro-inflammatory cytokine activation of CRH pathways forms the basis of the HPA axis overactivity in depression. Thus, this pathway represents a primary mechanism by which cytokines contribute to the development of depression (Müller and Ackenheil, 1998; Haddad et al., 2002; Leonard and Myint, 2009).

The interaction between cytokines and glucocorticoids during a stress response is complex (Figure 6). On one hand, stress can directly activate the peripheral immune system, and on the other hand, stress inhibits the peripheral immune response by activating the HPA axis and the sympathetic nervous system. Peripheral pro-inflammatory cytokines activate the release of brain cytokines, which then activate neuronal circuits and the HPA axis. Glucocorticoids inhibit the release or function of these pro-inflammatory cytokines, and they also inhibit the HPA axis through a negative feedback loop.

This brings us to the neuro-immune hypothesis of depression. In 1977, Bartrop et al. (1977) pioneered the concept of the psycho-neuroimmunology of depression. They were the first to hypothesize that severe psychological stress might produce measurable abnormalities in immune function, which could not be explained by hormonal changes. Since that time, studies have shown that MDD is often accompanied by chronic inflammatory changes (Leonard, 2005, 2006, 2007, 2014; Leonard and Myint, 2006a,b; Leonard and Myint, 2009).

An essential component in the neuro-immune hypothesis of depression is that patients with depression exhibit elevated levels of CRH. This neuropeptide orchestrates the stress-induced activation of the HPA axis (with the release of ACTH and cortisol), activates the locus coeruleus (behavioral component) (Valentino and Foote, 1988), and stimulates the sympathetic nervous system (and release of catecholamines) (Tsatsanis et al., 2007). Stress leads to the activation of macrophages in the blood and microglia in the brain, which results in the release of pro-inflammatory cytokines. This stress-induced release represents one of the primary mechanisms by which cytokines may contribute to the development of depression (Leonard, 2006, 2007, 2014). These findings supported the notion that chronic inflammation may be a central factor in the pathogenesis of MDD. Pro-inflammatory cytokines stimulate the production of neurotoxic substances (by stimulating the tryptophan-kynurenine pathway), induce specific inflammatory changes (iNOS and COX2 activation), and cause hypersecretion of cortisol. Cortisol inhibits protein synthesis, which reduces the production of anti-inflammatory and neurotrophic factors. The final result is the emergence of structural neuronal alterations, in particular, hippocampal neuronal cell loss. According to this hypothesis, depression leads to structural changes that can be considered neurodegenerative, and this condition may progress into Alzheimer's disease and other dementias. Serotonin may offer protection against this damaging influence. Serotonin appears to have a substantial impact on normal CNS development and function (Sodhi and Sanders-Bush, 2004). Serotonin acts as a growth factor during embryogenesis; serotonin receptor activity is a crucial part of the events that lead to changes in brain structure. In addition, the serotonergic system interacts with BDNF, which might explain why antidepressant drugs increase BDNF signaling (Castrén, 2004a,b). It has been suggested that antidepressant drugs have chronic effects associated with the repair, and possible reconstruction, of neuronal networks (Castrén, 2004a,b; Castrén et al., 2007). Antidepressants are known to enhance axonal and dendritic sprouting, and they are believed to stabilize synaptic contacts (Leonard, 2007). Thus, antidepressant drugs may protect neurons from detrimental changes by promoting repair and construction or reconstruction of neuronal networks.

The neuro-immune hypothesis on the pathogenesis of depression remains indefinite. Several possible theories can explain how activating the CNS immune system causes functional changes in the CNS. Our explanation of the genesis of a depressive disorder focuses on the inhibition of hippocampal function. This inhibition may result in improper stimulation or disinhibition of the amygdala and/or disinhibition of the connection with the medial habenula. In addition, cytokines have a profound influence on neuronal excitability. When these two events come together in the amygdala, a phenomenon termed “kindling” may occur. This brings us to the last hypothesis for the pathogenesis of depressive disorders.

## Kindling (neuroplasticity/neurogenesis) hypothesis

The hypothesis of illness progression is often referred to as a “kindling” hypothesis. This theory holds that, analogous to the relationship between kindling and convulsions, some stressors may produce increasing effects over time, which culminate in a full-blown affective episode (Post, 2007, 2010). For example, the more often unipolar or bipolar episodes occur, the more vulnerable one is to recurrences and the development of cognitive dysfunction (Post et al., 2012). Here, we take this concept a step further to provide a potential explanation for the pathogenesis of depression.

In experimental models, kindling is characterized by a progressive increase in neuronal activity, as a result of daily repeated applications of (subluminal) electrical stimuli, typically in limbic areas, such as the amygdala or the hippocampus (Post, 2004, 2007). The kindling model is used to study both the development of clinical seizure disorders (e.g., temporal lobe epilepsy) and the genesis of behavioral disorders (particularly bipolar mood disorders). Amygdala kindling involves once-daily, repeated stimulations of the amygdala (typically pulses with a 1-s duration) at intensities that are below the amygdala after-discharge threshold. At first, this treatment causes after-discharges that gradually increase in duration and complexity, and they begin to spread through the surrounding cortical regions. Then, when this stimulation is continued, full-blown motor seizures begin to occur, and thereafter, seizures start occurring spontaneously, in the absence of exogenous electrical stimulation. In the kindling hypothesis, we propose that a similar progression occurs in depressive disorders, where initial episodes are triggered by psychosocial stressors, but with enough repetition, they also begin to emerge spontaneously.

**Figure 7:**
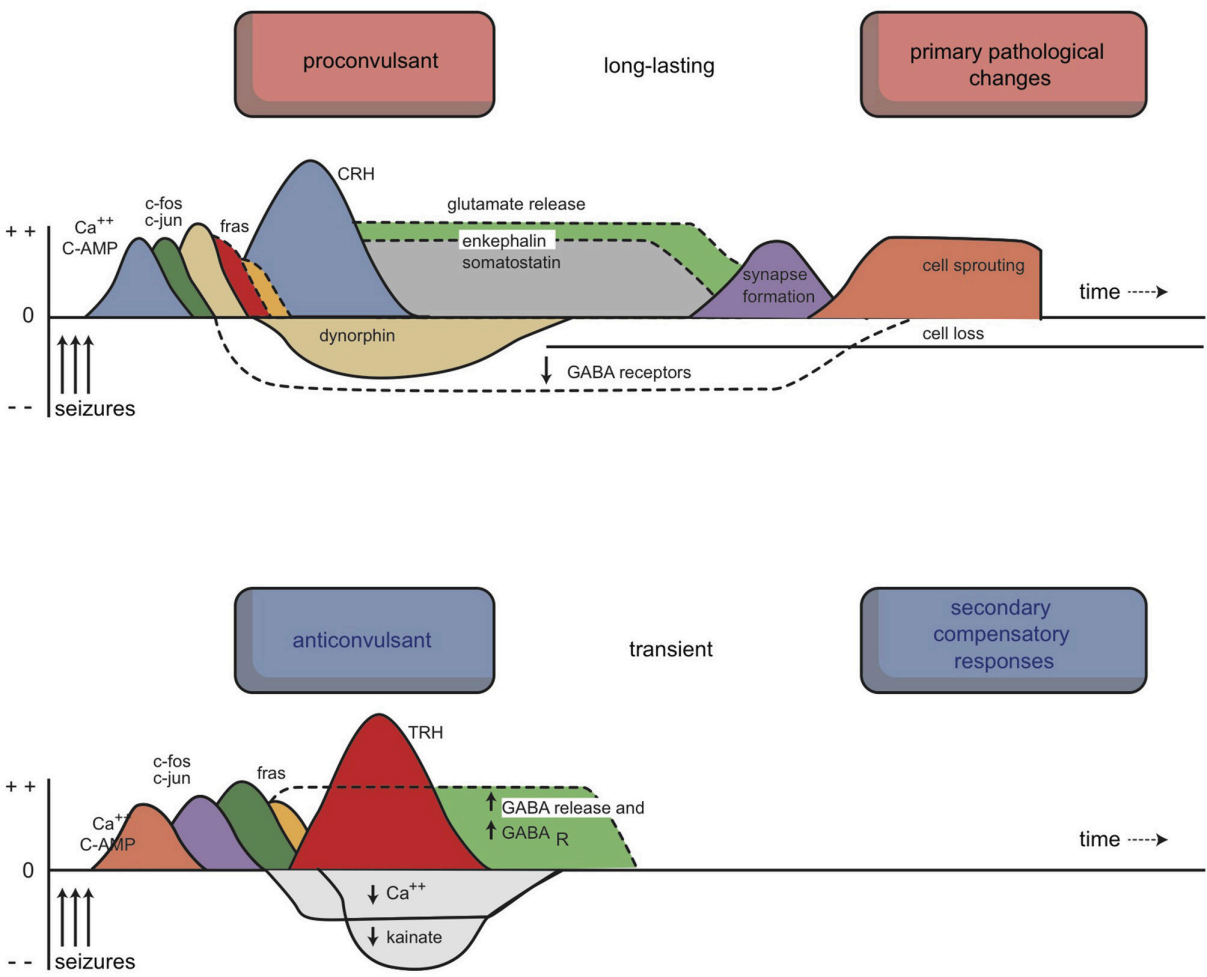
**Competing pathological and adaptive endogenous responses to kindled seizures**. Cellular components are shown for the (**upper panel**) proconvulsant and (**lower panel**) anticonvulsant biochemical (neuroplastic) reactions to a kindled seizure. Increased and decreased biochemical responses result in augmenting or inhibiting the ability of the neuronal system to display spontaneous electrophysiological activity (reproduced in an adapted form with permission from the author and publishers of Post, 2007).

Amygdala-kindling is accompanied by several biochemical changes involving classical neurotransmitters, neuropeptides, cytokines, and neurotrophic factors (Figure 7). Some of these changes are believed to represent a pathophysiological process that gives rise to the evolution of seizures and others are thought to reflect adaptive and compensatory changes that result in transient, anticonvulsant effects (Post, 2004, 2007). The latter changes comprise the induction or increased expression of thyrotropin-releasing hormone (TRH), cholecystokinin (CCK), neuropeptide Y (NPY), and benzodiazepine/GABA receptors. A key finding, which possibly links the kindling and the neuro-endocrine hypotheses, is the observation that CRH plays an important role in the evolution of kindled seizures. As an excitatory transmitter, CRH operates as the cardinal CNS transducer of stressful stimuli. Because the amygdala and hippocampus are rich in neurons that synthesize CRH or possesses CRH receptors, repeated stimulation of these receptors can result in limbic seizures (Brunson et al., 1998). In addition, corticosteroids are known to accelerate electrical amygdala kindling epileptogenesis (Karst et al., 1999; Taher et al., 2005; Kumar et al., 2007). This process may be an important mechanism for stress-induced facilitation of behavioral and mood disorders. Therefore, both neuronal (CRH) and humoral (steroid) factors appear to link the endocrine system to the limbic system.

The influence of cytokines and neurotrophic factors on depressive disorders is difficult to understand, which perhaps makes it a more intriguing issue. This issue is difficult even without considering the complex interactions among these factors. Their effects are often found to be biphasic and/or very complex in nature. This is true for the most intensely studied cytokines (IL-6, TNF-α, and IL-β) and for the less understood cytokines, e.g., BDNF. The first three cytokines (IL-6, TNF-α, and IL-β) increase the vulnerability to glutamate excitotoxicity (causing cell death), but they are also involved in neuroprotection against cell death (Bernardino et al., 2005a,b). The same is true for BDNF, which increases neuronal excitability and also has neuroprotective effects (Binder et al., 2001). Moreover, BDNF levels decrease in proportion to the severity of both depressive and manic episodes (Post, 2007). According to the model described by Post, BDNF appears to play an important role in both genetic and environmentally-induced vulnerability to the development of a (bipolar) mood disorder. Insufficient protection from kindling, due to a fall in BDNF levels, might induce the development of an affective episode (Post, 2007).

Returning to the kindling hypothesis, a few findings have supported the illness progression model. As described above, both corticosteroids and CRH are capable of enhancing the excitability of neurons in the corticoid amygdala, and consequently, lower the stimulation threshold. In various parts of the cortex, these compounds may be able to induce uncoordinated electrical activity, which finally results in a convulsion. The vulnerability to kindling in the amygdala was decreased by an infusion of BDNF (Osehobo et al., 1999; Barton and Shannon, 2005). In contrast, the other neurotrophic factors and cytokines may have the opposite effect. Moreover, because the amygdala lies deep inside the temporal lobe, it is difficult to demonstrate neurophysiologically in patients that the amygdala initiates the emotional response by producing uncoordinated electrical activity that leads to inappropriate, disproportional emotional reactions. However, BDNF activity was decreased in the amygdala of female patients with MDD (Guilloux et al., 2012). Therefore, although we lack hard evidence to support this hypothesis, the kindling hypothesis for illness progression may offer an explanation of how the mood disorder arises. Repeated psychosocial-stressful events can be considered a normal aspect of life. In normal conditions, the organism should be capable of adequately dealing with repeated stressors. However, in conditions where vulnerability is increased, either by genetic determination and/or by a transient physiological state, uncoordinated electrical activity may be induced, and the result is an inappropriate emotional response.

Based on this hypothesis, one would expect that biological measures with anticonvulsant effects in the amygdala would also have a therapeutic effect on mood disorders. This expectation was fulfilled with ECT, which is believed to exert therapeutic action by delivering potent anticonvulsant activity (Sanacora et al., 2003). ECT treatment raises the convulsive threshold substantially (Sackeim et al., 1987). It may be postulated that this anticonvulsant activity is necessary in the corticoid amygdala. However, not all anti-kindling treatments result in antidepressant effects. For example, several anticonvulsant drugs are primarily active in mania. Their efficacy in treating “bipolar” and “unipolar” depression is doubtful, at best. Only ECT appears to be effective in treating both depression and mania. Moreover, several effective TCA drugs (but not SSRIs) have the opposite effect; they enhance the vulnerability of the amygdala to kindling (Ago et al., 2007). This effect may correspond to their potential for inducing mania and “rapid cycling” in bipolar disorder, but it does not explain their therapeutic effects in depression. It supports the hypothesis that TCA drugs elicit their therapeutic effects in structures other than the hippocampal/amygdala complex. This brings into question whether ECT elicits antidepressant activity in the amygdala. Its effects on the hippocampus/amygdala may be primarily related to its anti-manic activity, and its antidepressive effects may occur elsewhere. For example, these latter effects could occur in the subgenual cingulate cortex (Seminowicz et al., 2004), which is part of the limbic cortico-striato-thalamo-cortical (CSTC) re-entry circuit that includes the NAcbS, which regulates the motivation to escape misery.

## Integration with our depression model

It can be concluded that neuroplastic changes seem to connect all five theories on the pathogenesis of depressive disorder. Chronic treatment with antidepressants of several different classes is known to influence the expression of neurotrophic factors, for example, antidepressants increase BDNF expression in both animals and patients (Nibuya et al., 1995; Sen et al., 2008); cortisol and several cytokines have profound neuroplastic effects; and the disturbed circadian cortisol rhythm may cause the biological clock mechanism to induce neuroplastic changes. All these changes might occur during chronic activation of the stress response, due to continuous stressful circumstances. In fact, it would make sense for the system to evolve in ways that improved the chance of survival under difficult living conditions. This evolution may be accomplished by memorizing a successful behavioral reaction and the circumstances that activated the reaction. This behavior can be memorized through an adaptation in the hardware that initiates and mediates the stress response.

Adaptation of reward-seeking and misery-fleeing behavior to changing circumstances is essential for maintaining life and for continuing the existence of the species. The earliest vertebrate ancestors of humans regulated these behaviors with a subcortical network that included the forebrain basal ganglia, forebrain habenula, and motor control centers in addition to monoaminergic regulation areas in the upper brainstem and hypothalamus (Robertson et al., 2014; Loonen and Ivanova, 2015). Therefore, it may be hypothesized that these subcortical structures also possessed the mechanics to adapt to major transformations in an animal's biotope. The mechanisms involved may have been conserved in humans, through the two NAcbC/NAcbS-containing CSTC re-entry circuits, which regulate the motivation for misery-fleeing and reward-seeking behaviors and through the amygdalar-hippocampal connections with the hypothalamus and midbrain. It should be recognized that, with evolution, the entire forebrain of the earliest vertebrates migrated up into the human amygdalar-hippocampal complex (Loonen and Ivanova, 2015; Loonen and Ivanova, submitted). This complex retains direct connections with the hypothalamic and midbrain control centers, but it also maintains indirect connections, via the medial and lateral habenula; and these latter structures have probably retained their regulatory role in humans.

The stress response is initiated by the amygdala, which receives stress inhibiting information from the PFC and the hippocampus. When these structures begin to malfunction, due to chronic stressful conditions, the stress response reaches an unusually high level, which results in a mismatch between misery-fleeing and reward-seeking behaviors. This mismatch continues as long as the individual cannot escape from the troubling circumstances. The inability to escape would lead to dysphoria, rumination, and negative expectations/hopelessness (due to high activity in the NAcbS-containing CSTC circuit) and anhedonia, lack of energy, and indecisiveness (due to low activity in the NAcbC-containing re-entry circuit). This impairment can be treated with psychotherapy, by increasing the abilities of the hippocampus and PFC to ease their hyperactive signaling. It can also be treated with biological treatments by interfering with the signal that activates the amygdala to induce the disproportionate stress response, and by directly inhibiting the NAcbS circuit.

The above scenario is supported by the fact that anyone can get depressed, when the burden of stressful circumstances is sufficiently intense and continuous. However, it does not explain why depression is so prevalent in the present era, to the extent that it may be the third leading cause of disability worldwide (Kessler, 2012). In our opinion, this high prevalence is partly due to overactive biological mechanisms that regulate behavioral adaptation to changes in environmental circumstances. This type of overactivity may arise from allostatic overload, due to developmental, ecological, social, and individual physical health influences (McEwen, 2012, 2015); from genetic and epigenetic vulnerability; from prolonged exposure time, due to increased life expectancy; from decreased selection pressure; and from redundant defensive activity, due to the absence of real threats. Consequently, we suggest that studies on the epidemiology, course, and causes of depression should not assume that depression is an illness; instead, depression should be considered a general form of reacting that indicates an impairment in the individual's adaptation mechanism. The likelihood of becoming depressed might depend on the individual's vulnerability to neuroplastic changes, which may be conferred by genetic factors, prior experiences, physical diseases, and toxic influences. These potential sources of vulnerability comprise suggestions for future research.

Certain components of our anatomical model require verification. For example, our model indicates the existence of re-entry circuits that regulate misery-fleeing and reward-seeking behaviors. To our knowledge, no study has shown that the fibers running from the thalamus to the subgenual cingulate cortex contribute to a cortical-subcortical re-entry circuit (Figure 2).

Moreover, the role of the insular cortex should be further elucidated. We have hypothesized that this phylogenetically ancient part of the cerebral cortex plays a critical role in perceiving feelings of pleasure (satisfaction) and happiness (euphoria) (Loonen et al., 2016). Indeed, the insular cortex was demonstrated to be involved in processing emotions, like anger, fear, happiness, sadness, or disgust, and it has been shown to display treatment-responsive changes in activity associated with different mood disorders (Nagai et al., 2007). However, the exact mechanism of becoming aware of these emotions remains unclear.

In addition, it may be of interest to identify the human limbic homolog of the habenula-projecting globus pallidus (GPh) of lampreys. The lamprey is the modern representative of theearliest vertebrate ancestors of humans (Loonen and Ivanova, 2015). In lampreys, the GPh regulates the activity of the lateral habenula, which in turn, controls the activity of the phylogenetic ancestor of the VTA (Robertson et al., 2014). In our opinion, the earliest vertebrate's striatum and pallium (primitive cortex) later became integrated with the hippocampal complex and amygdala. The output of the habenula to monoaminergic centers within the upper brainstem has been very well conserved during evolution (Aizawa et al., 2011; Velasquez et al., 2014; Antolin-Fontes et al., 2015). Similarly, an equivalent of the GPh may play a critical role in regulating the reward-seeking response. A good candidate for this GPh-equivalent might be a group of habenula-projecting neurons within the bed nucleus of the stria terminalis (Loonen and Ivanova, submitted).

The potential roles of the circuits described in our model can be investigated in neuroimaging studies, conducted before and after challenging these circuits with selective pharmacological and neurotrophic probes. However, because the habenula has a very limited size and complex structure, even a high-resolution fMRI approach offers limited possibilities for studying this structure. Also, animal studies can provide fruitful data, collected after systemic and local applications of substances. However, this research should begin by reinterpreting the many existing data, within the context of our model.

## Author contributions

AL developed these ideas during about 15 years in a series of consecutive lectures and educational courses. In 2015 this was during an educational CINP course in Tomsk, which was chaired by SI. She inspired him to write the current manuscript in a fruitful collaboration. Thus, both authors have made a substantial contribution to developing the conception and design of the manuscript.

### Conflict of interest statement

Nothing to declare for this specific study. AL received a speaker's fee and an unconditional research grant from Servier Pharma Netherlands. SI has nothing to disclose.
